# Diet-induced induction of hepatic serine/threonine kinase STK38 triggers proinflammation and hepatic lipid accumulation

**DOI:** 10.1016/j.jbc.2023.104678

**Published:** 2023-04-05

**Authors:** Priya Rawat, Shilpa Thakur, Surbhi Dogra, Kajal Jaswal, Budheswar Dehury, Prosenjit Mondal

**Affiliations:** 1 School of Basic Sciences, IIT Mandi, Mandi, India; 2 Bioinformatics Division, ICMR-Regional Medical Research Centre, Bhubaneswar, India

**Keywords:** fatty liver, high-fat diet, inflammation, STK38, hepatic insulin resistance, TBK1

## Abstract

Nonalcoholic fatty liver disease (NAFLD) is one of the most common liver diseases worldwide. Although the involvement of chronic overnutrition, systemic inflammation, and insulin resistance in the development of NAFLD is well-established, however, the associations among these remain to be elucidated. Several studies have reported that chronic overnutrition, such as excessive consumption of fats (high-fat diet, HFD), can cause insulin resistance and inflammation. However, the mechanisms by which HFD exerts inflammation and thereby promotes insulin resistance and intrahepatic fat accumulation remain poorly understood. Here, we show that HFD induces the expression of hepatic serine/threonine kinase 38 (STK38), which further induces systemic inflammation leading to insulin resistance. Notably, ectopic expression of STK38 in mouse liver leads to lean NAFLD phenotype with hepatic inflammation, insulin resistance, intrahepatic lipid accumulation, and hypertriglyceridemia in mice fed on a regular chow diet. Further, depletion of hepatic STK38 in HFD-fed mice remarkably reduces proinflammation, improves hepatic insulin sensitivity, and decreases hepatic fat accumulation. Mechanistically, two critical stimuli are elicited by STK38 action. For one stimulus, STK38 binds to Tank-Binding protein Kinase 1 and induces Tank-Binding protein Kinase 1 phosphorylation to promote NF-κβ nuclear translocation that mobilizes the release of proinflammatory cytokines and eventually leads to insulin resistance. The second, stimulus involves intrahepatic lipid accumulation by enhanced *de novo* lipogenesis via reducing the AMPK–ACC signaling axis. These findings identify STK38 as a novel nutrient-sensitive proinflammatory and lipogenic factor in maintaining hepatic energy homeostasis, and it provides a promising target for hepatic and immune health.

The global spread of high-fat diet (HFD), high-sugar diets, and sedentary lifestyles carry with it a sharp rise in nonalcoholic fatty liver disease (NAFLD) (1). The pathogenesis of NAFLD is a complex dysmetabolic process, following the “multiple-hit” hypothesis that involves hepatocyte excessive accumulation of triglycerides (TGs), insulin resistance, increased oxidative stress, chronic low-grade inflammatory response, and lipotoxicity. Although, several evidences suggest the association of HFD with NAFLD and insulin resistance with the release of proinflammatory mediators from the peripheral organs including the liver and adipose tissues, nevertheless, the mechanisms underlying NAFLD remain to be fully elucidated (2, 3, 4).

Inflammation is recognized as a critical pathophysiological factor in NAFLD. The release of proinflammatory cytokines from the liver and adipose tissue has been implicated in NAFLD and insulin resistance (5). Moreover, low-grade chronic inflammation has been suggested to induce insulin resistance in both humans and mice (6, 7, 8). Studies have shown an association between increased expression of inflammatory cytokines or chemokines MCP1, TNF-α, IL-6, IL-1β, etc., with insulin resistance. However, the causal factors and the molecular mechanism which drives HFD-induced inflammation still need to be fully understood. Current studies focus on elucidating the factors that drive hepatic inflammation in response to excessive consumption of HFDs. In search of an unconventional target that may be the putative node between obesity and inflammation, we found STK38. STK38, also known as NDR1 (nuclear dbf2p-related kinase 1), belongs to the NDR/LATS kinase family, one of the subclasses of the AGC group of serine/threonine kinase family. NDR kinase is known to regulate pattern recognition receptor-mediated innate immunity and cytokine-induced inflammation under bacterial infection (9). AGC group kinases are the evolutionary most conserved kinase family that consists of 14 subfamilies. The relevance of STK38 concerning metabolic disease is quite unexplored although its role has been implicated with cell cycle progression, apoptosis, autophagy, and transcriptional activity (10). All these observations prompted us to explore the role of STK38 in metabolic disorders.

In the present study, we identified STK38 as a positive regulator of NF-κβ signaling. Overexpression of STK38 in regular diet mice develop lean NAFLD-related metabolic disorders and showed increased inflammation and insulin resistance with enhanced intrahepatic lipid accumulation. Although there is a large population of obese people with NAFLD and type 2 diabetes, several reports suggest the global prevalence of NAFLD in lean or nonobese subjects is 5.1% and 12.1%; insulin resistance can occur even in nonobese individuals (11, 12, 13). Moreover, the prevalence rate of NAFLD in Asian lean individuals is very high and varies from 12.6 to 27% of lean individuals (14). Despite different etiologies, the progression of NAFLD in the lean individual is the same as in obese individuals. The clinical profile of lean NAFLD individuals shows high TG, fasting plasma glucose level, cholesterol, and less insulin sensitivity compared to healthy subjects with lobular inflammation and hepatocyte ballooning, similar to our observation of RD+AdSTK38 mice (15, 16). We further show that mice with hepatic overexpression of STK38 reduce T172 phosphorylation of α subunit of AMPK. In parallel, phosphorylation of ACC at S79 was reduced, which was consistent with increased intracellular triacylglycerol and cholesterol contents. Moreover, hepatic STK38 depletion mice are protected against HFD-induced insulin resistance, hepatic inflammation, and NAFLD. To explore the underlying mechanism in the activation of NF-κβ, we found TBK1, a putative interacting partner of STK38, a known activator of NF-κβ. Thus, our study suggests that STK38 is a critical node of the lipogenic/immune axis and provides a potential therapeutic target to ameliorate inflammation and insulin resistance in lean and nonlean NAFLD. Taken together, we strongly advocate STK38 be a critical hub protein with great therapeutic potential in NAFLD.

## Results

### HFD induces hepatic STK38 expression

**Figure 1:**
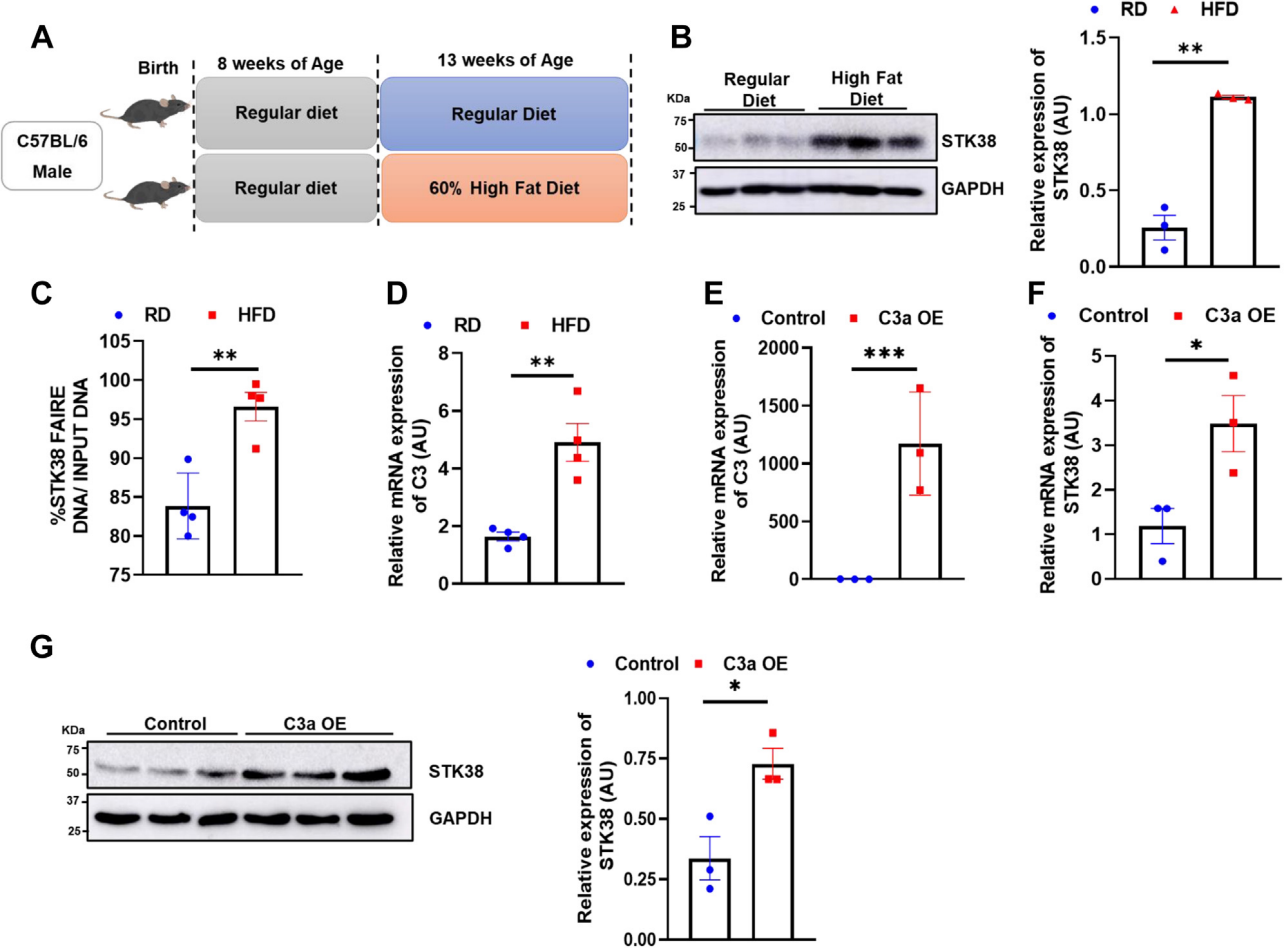
**HFD induces hepatic STK38**. *A*, schematic description of a dietary regimen of C57BL/6 male mice. *B*, qualitative and quantitative representation of STK38 protein level in the liver of RD and HFD mice. *C*, chromatin remodeling of STK38 is analyzed by FAIRE assay from the liver of RD and HFD. The percentage of active regulatory elements was calculated by dividing the QPCR signal of an isolated regulatory element by the Input DNA QPCR signal. *D*, expression of C3 in the liver RD and HFD mice at the transcription level. *E*, the transcript level of C3a was estimated after C3a overexpression in HepG2 cells for 48 h. *F*, the transcript levels of STK38 were analyzed after overexpression of C3a for 48 h in HepG2 cells. *G*, qualitative and quantitative analysis of protein level of STK38 after C3a overexpression in HepG2 cells. Mean±SEM. ∗*p* < 0.05, ∗∗*p* < 0.01,∗∗∗*p* < 0.001. C3, complement component 3; FAIRE, formaldehyde assisted isolation of regulatory elements; HFD, high-fat diet; RD, regular chow diet.

To explore the role of the NDR kinase family in metabolic disease, we chose STK38 as a representative. It was observed that hepatic STK38 protein expression significantly increased after male C57BL/6 mice were fed with HFD for 6 weeks, compared with that of mice maintained on a regular chow diet (RD) (Fig. 1, *A* and *B*). Further, we found SKT38 expression is significantly high under different conditions provided with high glucose, high insulin, insulin and glucose, pyruvate, and palmitate in HepG2 cells (Fig. S1*A*). Next, to investigate the reason underlying the increase of STK38 expression in HFD-fed mice liver, we perform formaldehyde assisted isolation of regulatory elements (FAIRE) assay to identify the sequence signature in open chromatin. After the isolation of the active regulatory element, we perform QPCR using STK38 promoter-specific primers. We found chromatin accessibility of STK38 is increased in the liver of HFD as compared to RD (Fig. 1*C*). Further, we explored the transcription factors that can bind to the promoter of the STK38 using a DNA pull-down assay (Table S1). Surprisingly, we found C3, a complement component protein binding to the promoter region of STK38. Further, hepatic *C3* mRNA expression was also significantly increased in HFD mice compared with that of mice maintained on RD (Fig. 1*D*). To confirm that C3 alone can induce STK38 expression, we cloned the activated domain of C3, *C3a* in pAAV-MCS and transfect it in the HepG2 cells. Overexpression of *C3a* was confirmed by RT-PCR (Fig. 1*E*). The transcript level of *STK38* was found high in C3a-transfected condition (Fig. 1*F*). Concurrently, the protein level of STK38 was also found significantly higher in C3a-transfected condition (Fig. 1*G*). This confirms C3a can modulate the expression of STK38. Together, these findings indicated HFD activates C3 which further induces the expression of STK38.

### Liver STK38 expression correlates with hepatic insulin resistance

**Figure 2:**
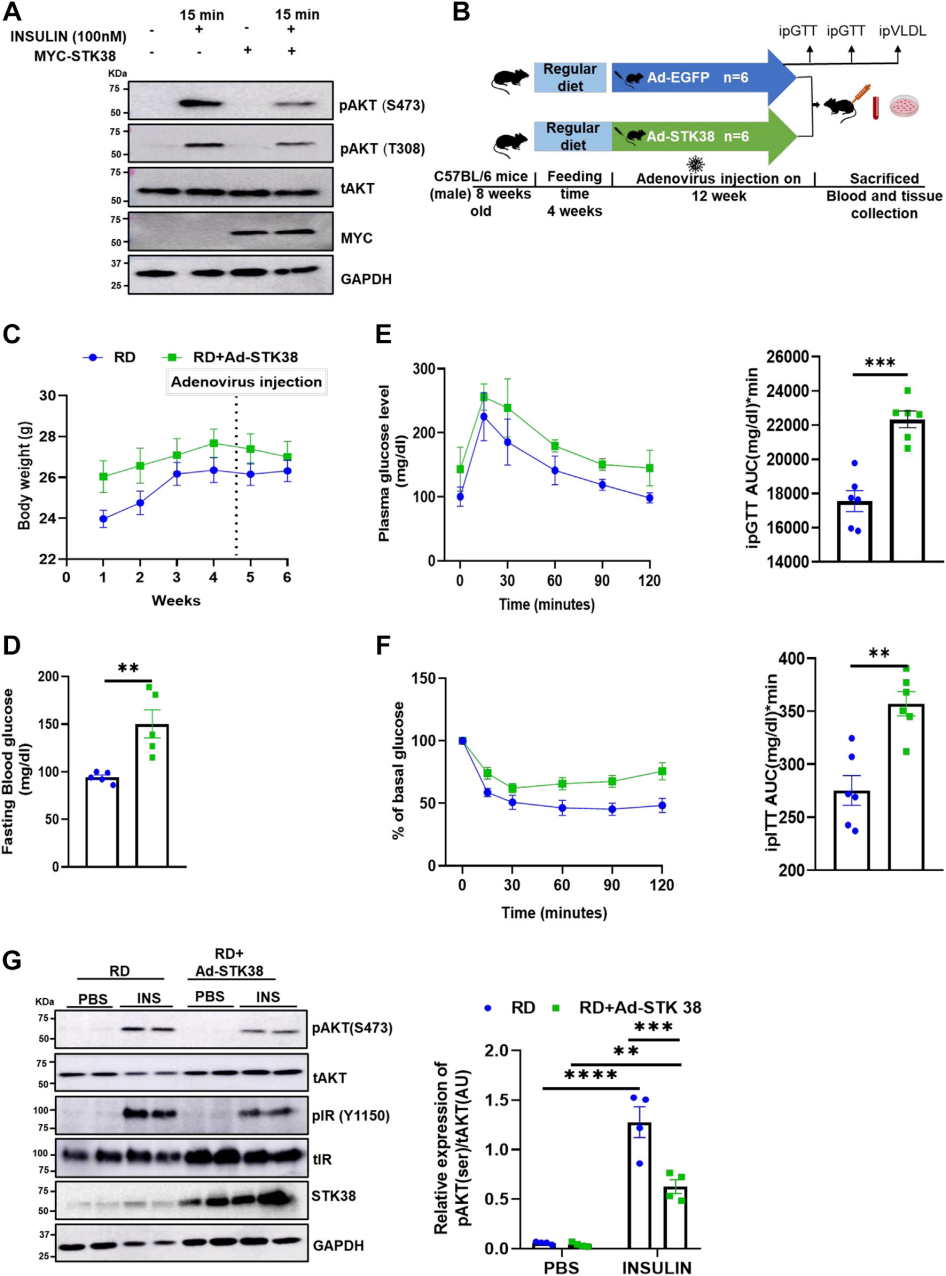
**STK38 induces hepatic insulin resistance**. *A*, STK38 is overexpressed in HepG2 cells for 48hrs and stimulated with 100 nM insulin for 15 min and analyzed for pAKT(S473) and pAKT(T308) protein levels by Western blot. *B*, schematic description of *in vivo* study. *C*, graphical representation of body weight. *D*, basal blood glucose in RD and RD+Ad-STK38 mice after 6 h of fasting. *E* and *F*, mice fasted for 6hrs. 2 g/kg glucose or 0.5IU/Kg insulin was injected intraperitoneally, and GTT and ITT were performed at 0, 15, 30, 60, 90, and 120 min and represented as AUC, respectively. *G*, qualitative representation of AKT phosphorylation at S473 and IR at Y1150, and quantitative representation of AKT phosphorylation in the liver of PBS and insulin-injected RD and RD+Ad-STK38 mice. Mean±SEM. ∗∗*p* < 0.01, ∗∗∗*p* < 0.001, ∗∗∗∗*p* < 0.001. ITT, insulin tolerance test; RD, regular chow diet.

Prolonged HFDs are closely associated with insulin resistance and inflammation. To determine the role of STK38 in hepatic insulin signaling, STK38 is overexpressed in HepG2 cells and its downstream effects were analyzed. Phosphorylation of AKT (S473), and AKT (T308), was measured as a surrogate for insulin resistance. Interestingly, STK38 overexpression reduces the phosphorylation of AKT at both S473 and T308 residues (Figs. 2*A* and S2*A*), while STK38 knockdown induces insulin sensitivity in HepG2 cells. (Fig. S2*B*). To confirm these observations *in vivo*, 8-weeks-old mice were kept on RD for 4 weeks, and STK38 overexpression was done using adenovirus via tail vein injection (Fig. 2*B*). The overexpression was confirmed by Western blotting (Fig. S2*C*). There was no significant difference in the body weight of the RD+Ad-STK38 mice as compared to the RD+Ad-EGFP mice (Fig. 2*C*). The liver of the RD+Ad-STK38 mice was found to be discolored (Fig. S2*D*). Moreover, the RD Ad-STK38 mice showed higher fasting blood glucose with impaired glucose tolerance test (Fig. 2, *D* and *E*). To examine whether this impaired glucose homeostasis is due to reduced systemic insulin sensitivity, we performed intraperitoneal insulin tolerance test (ipITT). In contrast to RD+Ad-EGFP mice, RD+Ad-STK38 mice showed a reduced insulin-dependent response (Fig. 2*F*). Further, to determine the impact of STK38 overexpression on hepatic insulin signaling in RD-fed mice liver, insulin (0.5 IU/kg body weight) or PBS was injected intraperitoneally into fasted mice of all experimental groups. At 10 min, tissues were collected, and the protein expression of pAKT (S473) and IR (insulin receptor) phosphorylation at Y1150 was analyzed in the liver. Liver from RD+Ad-EGFP mice showed significantly increased levels of pAKT with enhanced pIR upon insulin injection (Fig. 2*G*), demonstrating that intraperitoneal insulin administration achieves circulating insulin levels at 10 min adequate for inducing signaling in liver tissue. However, no significant increase in pAKT (S473) and pIR (Y1150) was observed in response to insulin from RD+Ad-STK38 liver lysates; this confirmed disrupted hepatic insulin signaling in RD+Ad-STK38 mice liver. Taken together, these results suggest that an elevated expression of hepatic STK38 may exacerbate hepatic and systemic insulin resistance.

### STK38 potentiates NF-κβ transactivation via TBK1

**Figure 3:**
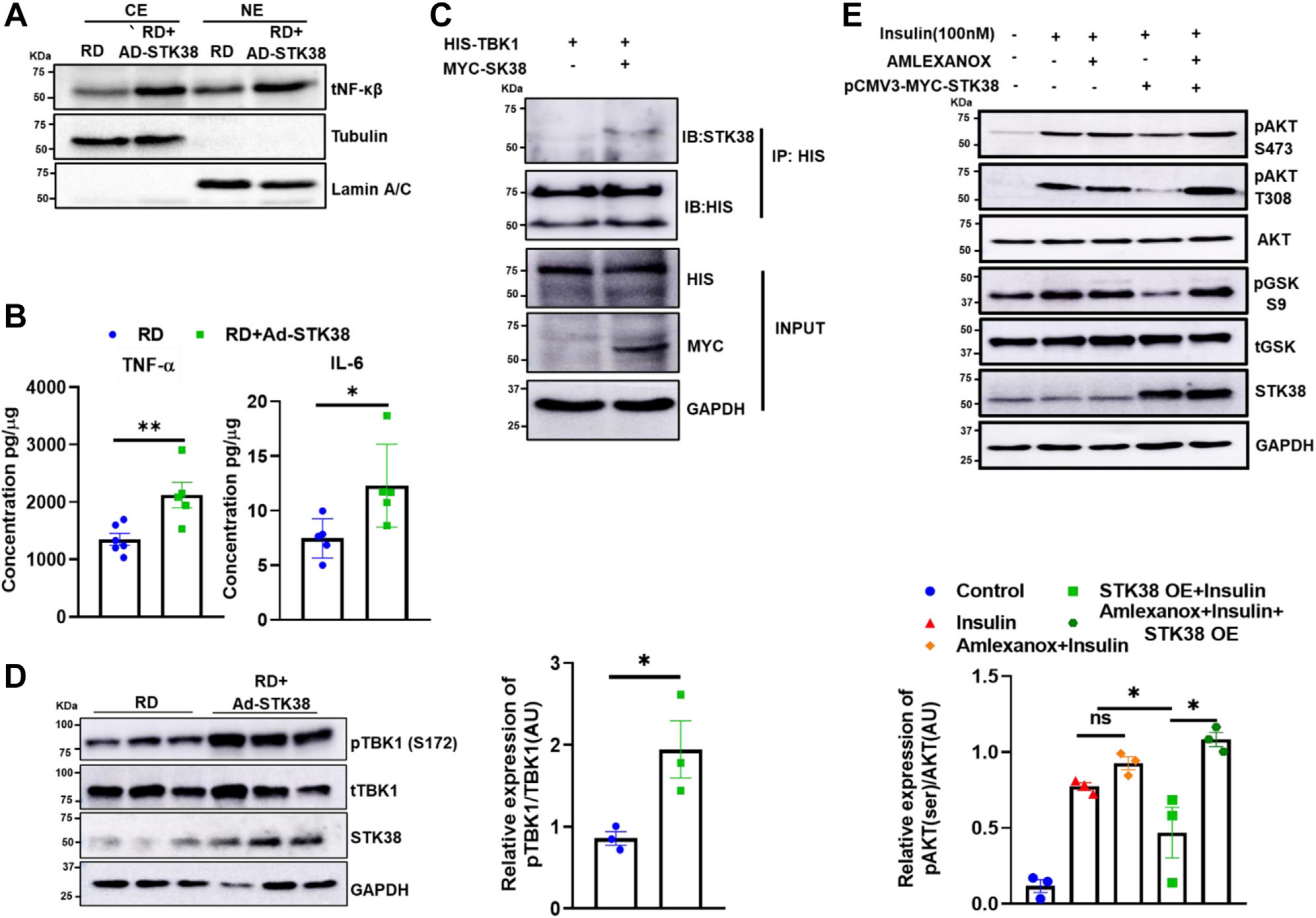
**STK38 potentiates NF-κβ transactivation via TBK1.***A*, qualitative representation of the level of NF-κβ in nuclear/cytosolic extract of RD and RD+Ad-STK38 mice liver tissue. *B*, the level of proinflammatory cytokines TNF-α and IL-6 in the liver of RD and RD+Ad-STK38 mice were estimated by using Bioplex 200. *C*, physical interaction between STK38 and TBK1 was analyzed by CO-IP. Both Myc-STK38 and His-TBK1 were cotransfected in HepG2 cells, and IP was performed using anti-his antibody and analyzed using Western blot. *D*, Western blotting was performed using a liver lysate of RD and RD+Ad-stk38 mice, and relative expression of pTBK1 was quantified using ImageJ. *E*, pharmacological inhibition of TBK1 rescued insulin resistance. STK38 was overexpressed in HepG2 cells and treated with 100 μM Amlexanox for 9 h before 48 h of incubation. Cells were stimulated with 100 nM insulin for 15 min, and AKT and GSK3β phosphorylation was analyzed by Western blotting and quantified by using ImageJ. Mean ± SEM. ∗*p* < 0.05, ∗∗*p* < 0.01. RD, regular chow diet.

Inflammation is associated with the pathogenesis of insulin resistance. Next, we wanted to determine whether reduced insulin sensitivity is associated with increased inflammation. To find the association between STK38-mediated occurrence of insulin resistance with inflammation, we investigated NF-κβ′s subcellular localization. NF-κβ is one of the known transcription factors known to induce increased transcription of proinflammatory cytokines. We overexpressed STK38 in HepG2 cells and performed immunocytochemistry for NF-κβ and found enhanced NF-κβ localization into the nucleus as compared to the control (Fig. S3*A*). Similarly, immunoblotting of subcellular-fractionated liver lysates revealed a significant rise in the abundance of NF-κβ protein in the nuclear lysate of RD+Ad-STK38 mice group as compared to RD+Ad-EGFP (Fig. 3*A*). Nuclear localization of NF-κβ p65 subunit is required for the optimal p65-mediated transactivation potential of NF-κβ. Thus, next, we accessed the proinflammatory cytokines in the liver of RD+Ad-EGFP and RD+Ad-STK38 mice, and we found a significant increase in the level of IL-6 and TNF-α from RD+Ad-STK38 mice (Fig. 3*B*).

To decipher the reason behind the activation of NF-κβ, we investigated the interacting partners of STK38 using string-db.org, and we found TBK1 as one of the interacting partners of STK38 (Fig. S3*B*). TBK1 has already been implicated with inflammation, and its activation is known to activate NF-κβ. To verify *in silico* data, we performed Co-IP, and STK38 was found to be physically interacting with TBK1 (Fig. 3*C*). Further, we performed a docking study using ClusPro, HADDOCK, and PRODIGY to identify the residues of STK38 interacting with TBK1 and found T444 residue of STK38 interacting with F45 and R47 (Fig. S3, *C*–*E* and Tables S2 and S3). To validate if T444 is important for the interaction of STK38 with TBK1, we mutated STK38 T444 residue to alanine and performed Co-IP, and we found STK38 is not able to interact with TBK1 (Fig. S3*F*). Also, binding energy analysis using PRODIGY showed STK38 T444 formed two crucial hydrogen bonds with F45 and R47 of TBK1 and one more hydrophobic contact with F45. However, when we mutated the STK38 WT with STK38^T444A^ mutant, we found a loss of hydrogen bonds and an increased deltaG (Table S4). This confirms site T444 residue is important for the interaction of STK38 and TBK1. Moreover, the activity of TBK1 is regulated by the phosphorylation at the S172 residue. Therefore, to inspect if STK38 is activating TBK1 via phosphorylation, we evaluate the phosphorylation of TBK1 at S172 in the liver tissue of STK38-overexpressing mice and we found enhanced TBK1 phosphorylation in STK38-overexpressing mice in contrast to control mice (Fig. 3*D*). Further, we investigated if TBK1 pharmacological inhibition using Amlexanox can rescue the insulin resistance in STK38-overexpressing HepG2 Cells. We analyze ATK signaling after the treatment of 100 μM Amlexanox in STK38-overexpressing HepG2 cells. We observed STK38 overexpression showed blunted insulin signaling axis as found by reduced pAKT (S473), pAKT (T308), and pGSK (S9). Whereas, pharmacological inhibition of TBK1 abrogated STK38-mediated attenuated insulin signaling (Figs. 3*E* and S3*G*). Together, STK38-induced reduction in the hepatic insulin signaling axis was blunted in Amlexanox-treated cells. These observations recommended TBK1 to have a pivotal role in STK38-mediated insulin signaling modulation. This substantiates that activation of TBK1 by STK38 causes blunt insulin signaling via transactivation of NF-κβ.

### STK38 exacerbates intrahepatic lipid accumulation in RD mice

**Figure 4:**
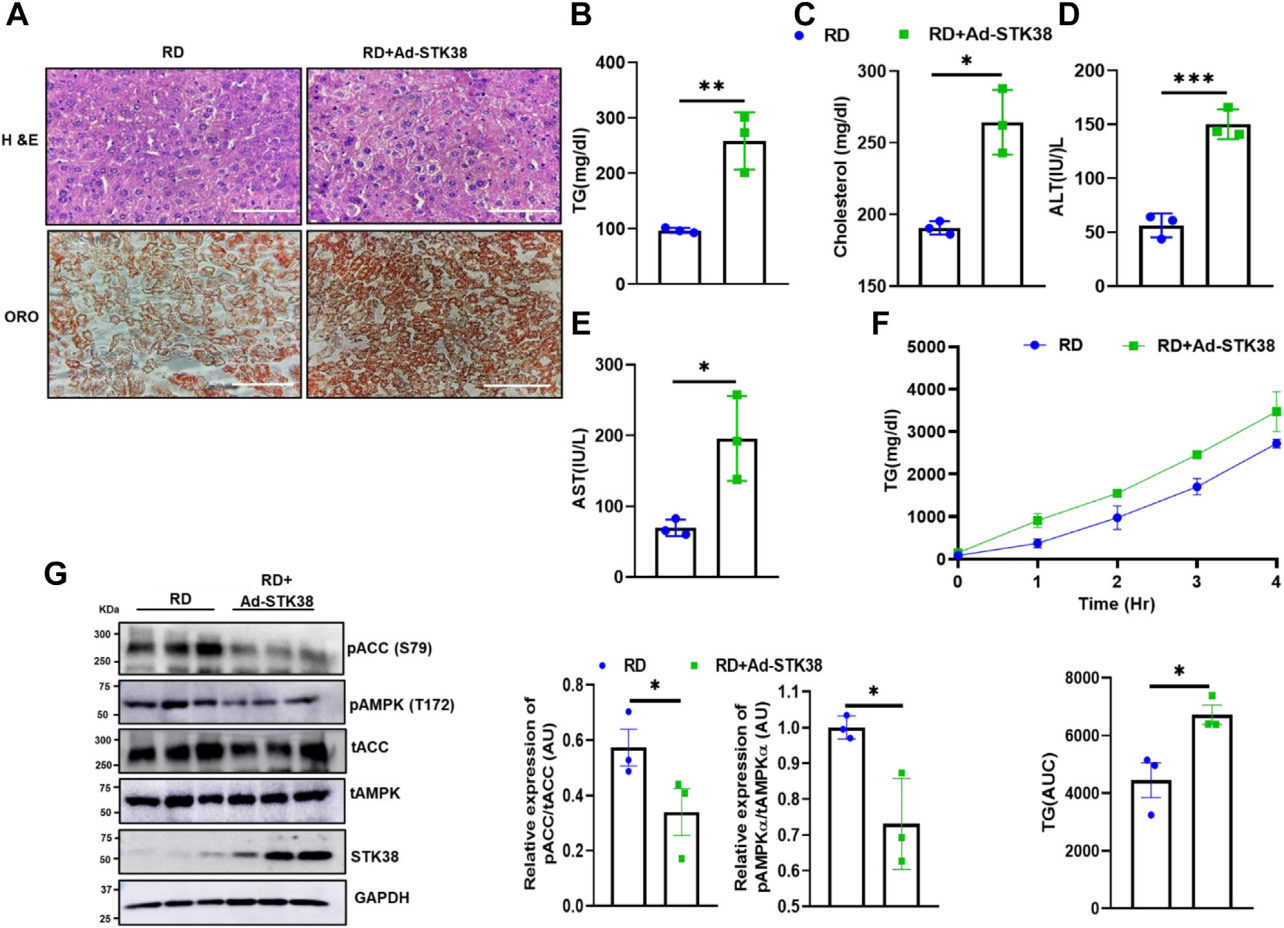
**STK38 exacerbates intrahepatic lipid accumulation.***A*, H&E and ORO staining of RD and RD+Ad-STK38 (40× magnification, scale bars represent 100 μm). *B*–*E*, serum TG, cholesterol, ALT, and AST level, respectively. *F*, TG content in plasma and secretion rate after intraperitoneal injection of poloxamer. *G*, STK38-mediated reduced phosphorylation of AMPK and ACC was analyzed by Western blot and quantified using ImageJ. Data expressed as Mean ± SEM. ∗*p* < 0.05, ∗∗*p* < 0.01, ∗∗∗*p* < 0.001. RD, regular chow diet; TG, triglyceride.

Accumulation of lipids in hepatocytes has a strong interconnection with insulin resistance. To dissect the impact of STK38 on lipid metabolism, we performed H & E staining of liver sections from Ad-EGFP– and STK38-overexpressing mice, and we found enhanced lipid accumulation in the liver of RD+Ad-STK38 mice. ORO staining also demonstrated an increased number and size of lipid droplets (Fig. 4*A*).

Next, we analyze the biochemical markers for liver function. The serum cholesterol and TG level were found to be high in STK38-overexpressing mice (Fig. 4, *B* and *C*). Further, the level of alanine aminotransferase and aspartate aminotransferase enzyme was also markedly high in RD+Ad-STK38 group (Fig. 4, *D* and *E*). To extend the finding of STK38 overexpression in lipid metabolism, we performed very low density lipoprotein- triglyceride secretion in mice by injecting poloxamer. Under these conditions, circulating TG is mainly derived from the liver. We found markedly high systemic TG in RD+Ad-STK38 group in contrast to RD (Fig. 4*F*).

Reports suggest that hepatic *de novo* lipogenesis (DNL) is an important contributor and regulator of HFD-induced hepatic lipid accumulation (16). DNL is regulated by the activation of AMPK in the liver (17). Thus, we aimed to study the effect of STK38 overexpression on AMPK activation. We observed that RD+Ad-STK38 mice with hepatic overexpression of STK38 reduce T172 phosphorylation of the α-subunit of AMPK compared to RD+Ad-EGFP group. In parallel, ACC(S79) phosphorylation was also reduced (Fig. 4*G*), which was consistent with enhanced intracellular TGs and cholesterol contents (Fig. 4, *B* and *C*). Consistent with the study, TBK1 and C3a overexpression in HepG2 cells also showed lipid accumulation (Fig. S4*A*). Integrating these results, we can conclude that STK38 contributes to hepatic lipid accumulation even in RD-fed lean mice.

### STK38 knockdown rescued HFD-induced impaired glucose tolerance and hepatic insulin resistance

**Figure 5:**
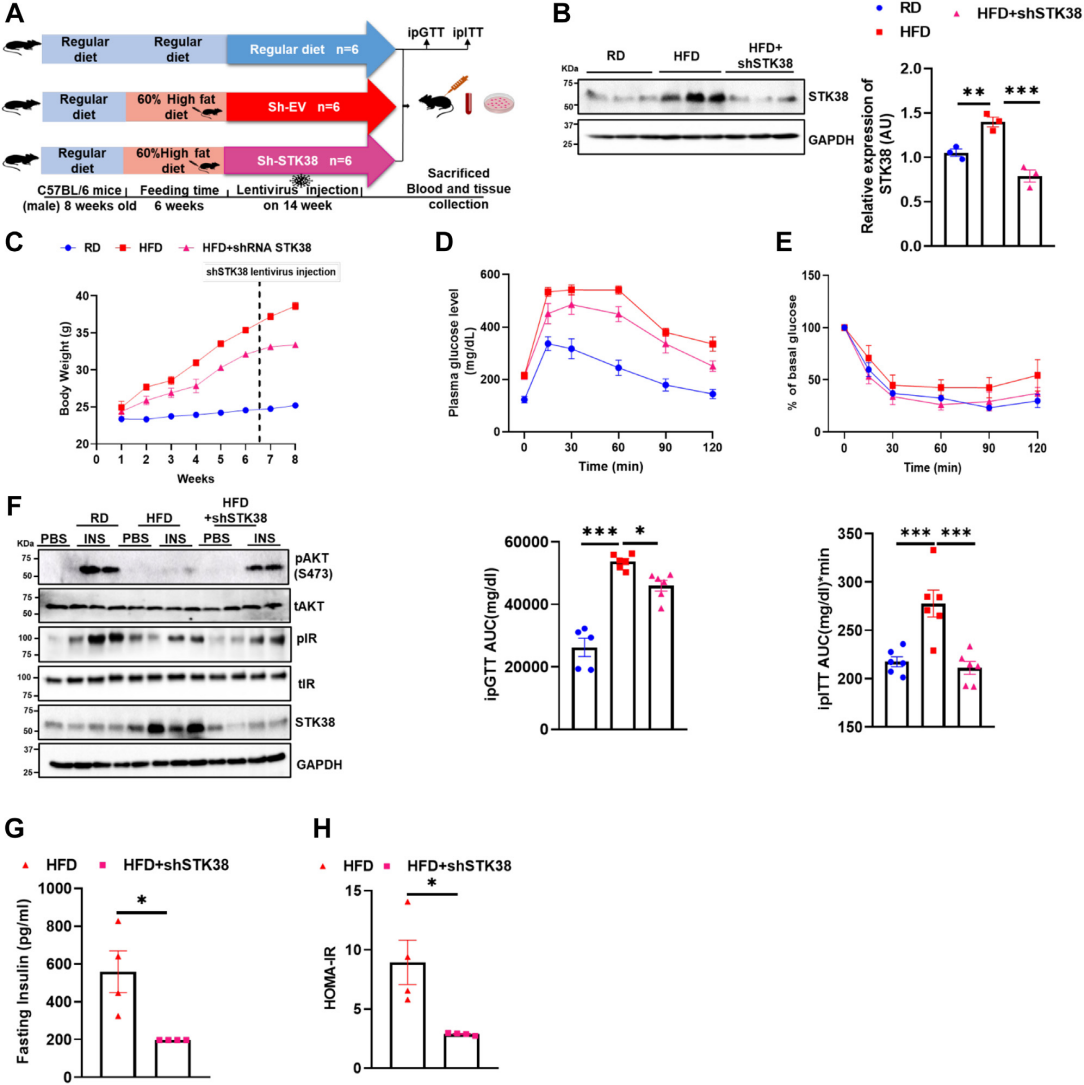
**STK38 knockdown in HFD mouse model improves high-fat diet–induced insulin resistance.***A*, schematic description of a dietary regimen of C57BL/6 male mice for STK38 knockdown. *B*, qualitative and quantitative representation of STK38 protein level in RD, HFD, and HFD+shRNA STK38 mice. *C,* graphical representation of body weight of RD, HFD, and HFD+shSTK38 mice. *D* and *E*, mice fasted for 6 h. 2 g/kg glucose or 0.5IU/Kg insulin was injected intraperitoneally, and GTT and ITT were performed at 0, 15, 30, 60, 90, and 120 min and represented as AUC respectively. *F*, qualitative representation of AKT phosphorylation at S473 and IR at Y1150 in the liver of RD, HFD, and HFD+shSTK38 mice injected with PBS and insulin. *G*, fasting insulin level in HFD and HFD+shSTK38 mice. *H*, HOMA-IR in HFD and HFD+shSTK38. Mean±SEM. ∗*p* < 0.05, ∗∗*p* < 0.01, ∗∗∗*p* < 0.001. GTT, glucose tolerance test; HFD, high-fat diet; ITT, insulin tolerance test; RD, regular chow diet.

Next, we analyzed if STK38 knockdown in HFD can improve HFD-induced hepatic insulin resistance. We fed mice with RD and HFD for 6 weeks, and STK38 knockdown was done using shRNA lentivirus (Fig. 5*A*). We found a significant downregulation of STK38 in HFD+shSTK38 mice as compared to HFD mice (Fig. 5*B*). We observed no difference in the body weight of HFD and HFD+shSTK38 mice after 1 week of shRNA lentivirus injection (Fig. 5*C*). Moreover, STK38 knockdown HFD mice showed improved intraperitoneal glucose tolerance test (ipGTT) and ipITT as compared to HFD (Fig. 5, *D* and *E*). However, there was no change in the fasting blood glucose between HFD and HFD+shSTK38 mice (Fig. S5*A*). The liver of HFD+shSTK38 mice was less pale as compared to HFD mice (Fig. S5*B*) To explore if STK38 knockdown can rescue diet-induced hepatic insulin resistance, we explored the AKT phosphorylation at S473 in the liver tissue in insulin-stimulated mice and found enhanced AKT phosphorylation at S473 in STK38 knockdown mice as compared to HFD mice with increased insulin receptor at Y1150 residue (Fig. 5*F*). Fasting insulin levels of STK38 knockdown HFD mice were found low as compared to HFD (Fig. 5*G*). Also, HOMA-IR was found to be reduced in STK38 knockdown HFD mice (Fig. 5*H*). Taking it all together, our data strongly suggest that STK38 knockdown can ameliorate diet-induced impaired glucose tolerance and hepatic insulin resistance.

### STK38 knockdown attenuates HFD-mediated hepatic inflammation and lipodystrophy

**Figure 6:**
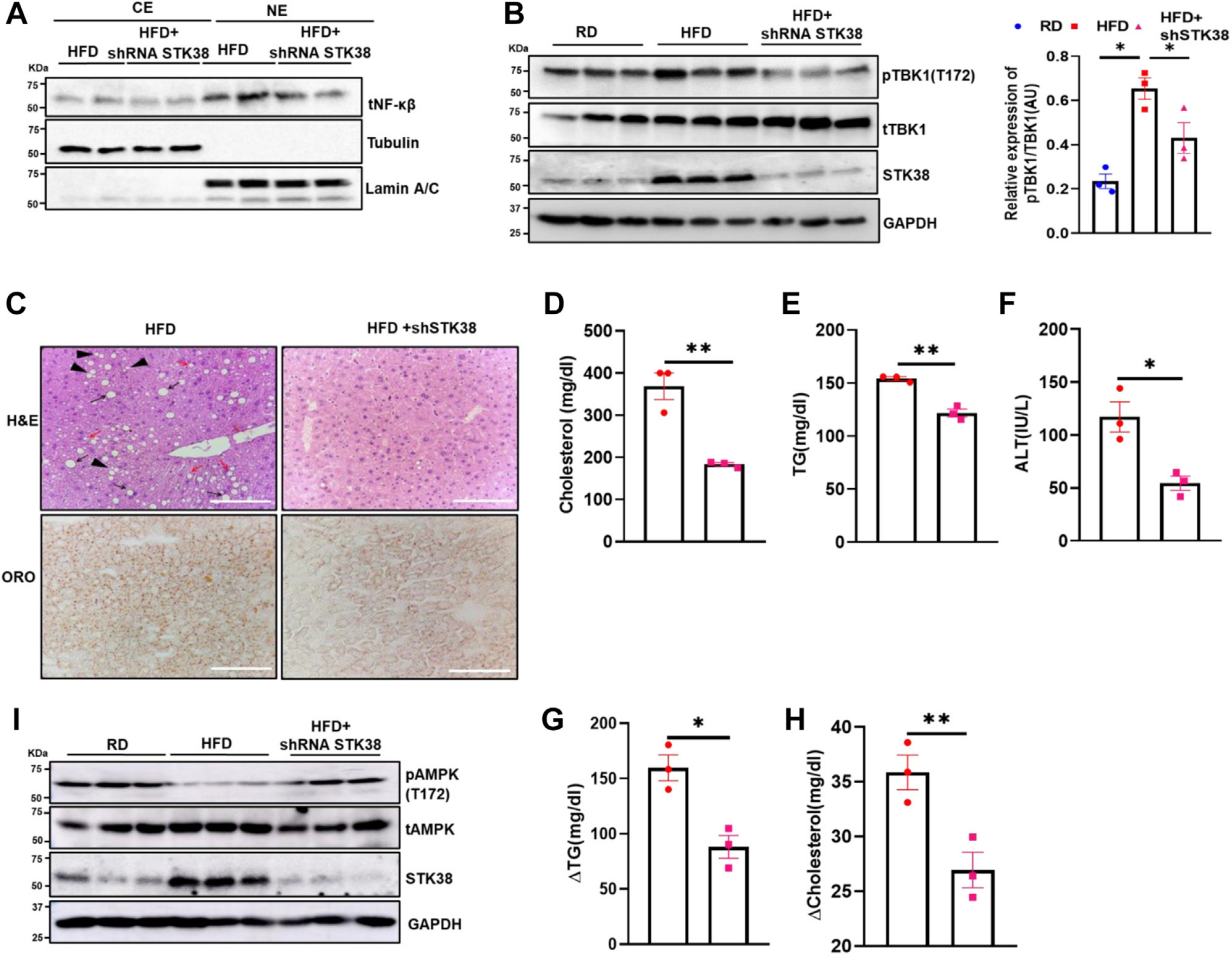
**STK38 knockdown attenuates high-fat diet–mediated hepatic inflammation and lipodystrophy.***A*, qualitative representation of the level of NF-κβ in nuclear/cytosolic extract of HFD and HFD+shRNA-STK38 mice liver tissue. *B*, STK38-mediated phosphorylation of TBK1 at S172. Western blotting was performed using a liver lysate of HFD and HFD+shRNA-STK38 mice, and relative expression of pTBK1 was quantified using ImageJ. *C*, images of H&E showing macrovesicular steatosis (signet ring cell, marked with *black arrow*), microvesicular steatosis (*red arrow*), and multiple vacuoles inside one cell (*black arrowhead*) and ORO staining in HFD and HFD+shRNA-STK38 liver tissue (40× magnification, scale bars represent 100 μm). *D*–*F*, serum cholesterol, TG, and ALT levels, respectively. *G* and *H*, hepatic cholesterol and TG level. *I*, qualitative representation of AMPK phosphorylation in the liver of RD, HFD, and HFD+shSTK38 mice. Mean ± SEM. ∗*p* < 0.05, ∗∗*p* < 0.01. HFD, high-fat diet; RD, regular chow diet; TG, triglyceride.

Our previous observations motivated us to study the impact of STK38 knockdown on HFD-induced hepatic inflammation and hepatic lipid accumulation. Thus, we assessed the cellular localization of NF-κβ in STK38 knockdown mice. Immunoblotting of subcellular fractionated liver lysates revealed a significant reduction in the abundance of NF-κβ protein in the nuclear lysate of HFD+shSTK38 mice group as compared to HFD+sh-EV mice liver (Fig. 6*A*). On a similar line, TBK1 phosphorylation at S172 residue in STK38 knockdown mice has also reduced significantly in contrast to HFD (Fig. 6*B*). Further, we performed immunohistochemistry for F4/80 from the liver tissue to analyze the macrophage infiltration in the liver. We observed lesser macrophages in STK38 knockdown HFD mice than HFD mice (Fig. S5*C*). In addition to this, we performed tunel assay and found reduced cell death in HFD+shSTK38 as compared to HFD mice (Fig. S5*D*). This data concludes that STK38 knockdown in an HFD dampens HFD-induced hepatic inflammation and hepatic cell death. Furthermore, we explored if STK38 knockdown can attenuate HFD-mediated lipodystrophy. We perform H&E and ORO staining from the liver tissue of HFD and HFD+shSTK38 mice. We found reduced lipid droplets in the liver of STK38 knockdown mice as compared to HFD mice (Fig. 6*C*). Moreover, we found that STK38 knockdown can reduce circulating TG as well as cholesterol levels in serum (Fig. 6, *D* and *E*) with reduced alanine transaminase levels (Fig. 6*F*). Furthermore, we found reduced hepatic TG and cholesterol levels in the STK38 knockdown mice as compared to HFD mice (Fig. 6, *G* and *H*). Taking cues from STK38 as a critical node of the lipogenic axis, we wanted to decipher the pathway involved in the regulation of hepatic DNL by STK38. We investigated the AMPK activation in response to STK38 knockdown in HFD mice. Immunoblotting of liver lysates revealed a significant reduction in the phosphorylation of AMPK at S172 residue of the HFD mice group as compared to RD, while phosphorylation of AMPK at S172 levels rises significantly after hepatic depletion of STK38 in the HFD group (Figs. 6*I* and S5*E*). This substantiates that STK38 knockdown attenuates the activation of TBK1, thus, inactivating NF-κβ and its localization into the nucleus-inhibiting HFD-induced hepatic inflammation and hepatic lipid accumulation. Taken together, these observations strongly suggest STK38 be a critical protein to modulate the proinflammatory/lipogenic axis via TBK1-NF-κβ and AMPK axis, respectively.

## Discussion

The present study aimed to understand the mechanism of how HFD exacerbates hepatic inflammation and insulin resistance and alters hepatic lipid metabolism. Despite the several evidence that excessive consumption of HFD and insulin resistance is associated with chronic low-grade inflammation and NAFLD, the node connecting these ends is still missing. Therefore, our findings establish that STK38 could be the node between high calorific diet–induced inflammation and intrahepatic lipid accumulation via the STK38–TBK1 signaling axis. TBK1 is a serine/threonine kinase that regulates the noncanonical NF-κβ activation pathway (18, 19, 20, 21, 22, 23).

Several studies have implicated the role of TBK1 in metabolic diseases, including NAFLD and type 2 diabetes. Its inhibition has been shown to improve obesity-driven metabolic dysfunction (19, 20, 21). TBK1 has a key phosphorylation site, S172, necessary for its activation and functioning. However, the molecular mechanism of phosphorylation and activation of TBK1 is not fully understood (22, 23).

Using complementary *in vitro, in silico, and in vivo* experiments, we elucidated the mechanism of HFD-mediated systemic inflammation by inducing hepatic STK38 expression.

Here, we identify how the protein expression of STK38 is increasing in high-nutrient conditions. Our study reveals the expression of STK38 in the liver of an HFD is inappropriately enhanced by C3. We found that C3 binds to the promotor region of STK38. Several studies have implicated the role of C3 in metabolic diseases, including insulin resistance, liver dysfunction, and type 2 diabetes. It has been found that systemic protein level C3 is found to be high in diabetic patients and NAFLD (24, 25). C3 consists of two chains, alpha and beta (C3α and C3β, respectively), connected by a disulfide bond. Activation of C3 causes proteolytic cleavage of C3A into C3a and C3b (26, 27). However, one study has reported the nonproteolytic activation of C3 by phospholipid vesicle (28, 29). It has been reported before that C3 binds to DNA and modulates chromatin remodeling (30). Consistent with the existing reports, we found activated C3a translocates into the nucleus and binds to the promoter region of STK38 and modulates chromatin remodeling thus increasing the expression of STK38 in an HFD.

Recent studies have implicated the role of STK38 in inflammation, but the relevance concerning the metabolic disease is quite unexplored (10, 31). Our study broadens the role of STK38 with pathophysiological implications for hepatic insulin resistance and lipid accumulation. The study demonstrated that overexpression of STK38 causes blunt hepatic insulin signaling in both *in vitro* and *in vivo* models. STK38-overexpressed mice showed high fasting blood glucose compared to regular diet mice with impaired glucose and insulin tolerance tests. However, there was no gain in body weight in the STK38-overexpressing RD mice relative to control RD mice. Furthermore, we found that STK38 mice showed increased hepatic inflammation with increased TNF-α and IL-6 expression and enhanced NF-kβ localization into the nucleus.

Our observation prompted us to investigate how STK38 is regulating NF-κβ activation and modulates hepatic inflammation. Through text mining and string.org database, we generated the interactome map of STK38 and found TBK1 could be the potential interacting partner of STK38. TBK1 has already been associated with inflammation, and its activation is known to activate NF-κβ (32). We found STK38 physically interacts with TBK1 causing its phosphorylation at S172 residue and its activation. Activated TBK1 was reported to phosphorylate Iκβ causing the dissociation of Iκβ from NF-κβ and nuclear translocation of NF-κβ (33). NF-κβ translocates into the nucleus and activates the transcription of proinflammatory cytokines (34, 35). The effect of STK38 on systemic inflammation is mediated through direct binding and enhanced phosphorylation of TBK1. Reduced hepatic STK38 and/or pharmacological inhibition of TBK1 results in attenuating inflammation and restoring hepatic insulin sensitivity *in vivo* and *in vitro*. Reduced STK38 (HFD+shRNA STK38) ameliorates HFD-induced impaired glucose tolerance, insulin resistance, and hepatic steatosis in HFD mice mainly by controlling hypophosphorylation of TBK1. Based on the observations, we propose STK38 increase the expression of some inflammatory cytokines via TBK1 signaling pathways, both of which are involved in the pathogenesis of hepatic as well systemic insulin resistance by interfering with insulin signaling and action.

Given the fact that the worldwide prevalence of rate of NAFLD is strongly linked to diabetes and insulin resistance, we were keen to assess the effect of STK38 on lipid homeostasis. We found that STK38 overexpression in RD mice showed increased systemic as well as hepatic lipid accumulation. In our current study, we observed the systemic cholesterol level was high, with a high TG level in STK38-overexpressing mice. The TG secretion has two primary sources, firstly, from the diet and, secondly, from the liver (in the form of VLDL). VLDL acts as a transport system of the excess energy in the form of TG to the extrahepatic tissues. Concordant with this, we found enhanced TG secretion in STK38-overexpressing mice in VLDL assay. In insulin-resistant state, enhanced FA secretion in serum causes enhanced delivery to the liver, instigating the high VLDL secretion. Also, in the insulin-resistant state, there is high circulating insulin, but the liver is unable to respond to it, which causes the liver to flinch to DNL, thus, causing enhanced VLDL secretion (36). This hypertriglyceridemia can further aggravate liver dysfunction. Moreover, there were high lipid droplets in the liver of STK38-overexpressing mice as confirmed by ORO and H&E staining. Additionally, we found reduced AMPK phosphorylation. Conversely, the knockdown of STK38 in the diet-induced insulin-resistant mice model improved GTT, and ITT with restored hepatic insulin sensitivity reduced hepatic inflammation, reduced macrophage infiltration, cell death, and lipodystrophy via inhibiting TBK1 phosphorylation and NF-κβ transactivation.

In HFD mice, the liver is exposed to two critical stimuli elicited by STK38 action. Firstly, intrahepatic lipid accumulation by enhanced DNL via reduced AMPK and ACC phosphorylation and second, TBK1 activation that induces expression and release of proinflammatory cytokines via NF-κβ transactivation in the liver. These observations propose a direct parallel and/or sequential role of hepatic STK38 in nutrient-induced inflammation and eventually driving the development of insulin resistance and NAFLD onset/progression. Further, the results assign a heretofore unknown role of hepatic STK38 in systemic metabolism.

Thus, our study highlights that STK38 can be a potential target to explore the missing link between those diet-induced molecular alterations that can induce hepatic inflammation and insulin resistance that can develop the lean fat phenotype. Chronic overnutrition emerged as a critical driver of inflammation and fibrosis in lean and nonlean NAFLD. Even though inflammation plays a key role in NAFLD pathogenesis, large clinical trials specifically targeting inflammatory pathways are still lacking. Our study suggests that STK38 is a critical node of the lipogenic/immune axis and provides a potential therapeutic target to ameliorate inflammation and insulin resistance in lean and nonlean NAFLD.

## Experimental procedures

### Mice

All experiments were approved by the Institutional Animal Ethics Committee at the Indian Institute of Technology Mandi, approved by the *Committee for Control and Supervision of Experiments on Animals (CPCSEA),* the Ministry of Environment and Forest, and the Government of India. C57BL/6 mice (Male, 8-week-old) procured from the animal house of IISER Mohali and acclimatized for 7 days at 23 °C and 50 to 60% humidity with 12 h light and 12 h dark cycle and free access to food and water. For the first study, mice were subjected to two groups for RD and an HFD. The RD group was fed with RD containing ∼14% calories from fat [protein 20%, fat 14.725%, carbohydrate 16.149%] (5L79 Lab diet), and HFD group was fed with a diet containing 60% calories from fat [protein 20%, fat 60%, carbohydrate 20%, and energy density 5.21% Kcal/g] (D12492, Research Diet). After 6 weeks on, RD and HFD mice were sacrificed and tissues and blood were collected. For the second study, mice were subjected to two groups RD (n = 6) and RD (n = 6) injected with STK38 adenovirus into the tail vein. For the third study, mice were subjected to three groups RD, HFD, and HFD injected with lentivirus carrying STK38 shRNA. The weight of the mice was recorded at the start of the experiment. ipITT and ipGTT were performed on the fourth and fifth day of overexpression and knockdown after fasting mice for 6 h (with free access to water). For ipGTT, mice were injected with 2 g/kg body weight glucose and for ipITT, mice were injected with insulin (0.50 IU/kg body weight) intraperitoneally, and glucose levels were measured at 15, 30, 60, 90, and 120 min after injection using Contour plus glucometer. The basal fasting glucose levels were measured before injection. The mice were sacrificed after at seventh day of injection. For each *in vivo* experiment, mice cohorts were randomized followed by the respective treatment of adenoviruses and lentiviruses for STK38 and shSTK38, respectively, via intravenous injections.

### Cell lines and culture treatment

Human hepatocellular carcinoma (HepG2) and HEK293T cell lines were cultured in Dulbecco’s modified Eagle’s medium high glucose (4.5 g/L) media supplemented with 10% fetal bovine serum and 1% penicillin-streptomycin and incubated at 37 °C and 5% CO_2_ incubator. To stimulate insulin signaling, HepG2 cells were treated with 100 nM insulin for 15 min and preceded for Western blot. For TBK1 inhibition, HepG2 cells were treated with 100 μM Amlexanox for 9 h and then treated with insulin to activate insulin signaling.

### FAIRE assay

FAIRE assay was done as previously done by Geresi *et al*. (37). Briefly, tissue was fixed using 4% paraformaldehyde for 4 h. Tissue was lysed using FAIRE buffer (10 mM Tris–HCl pH 8.0, 2% Triton X-100, 1% SDS, 100 mM NaCl, 1 mM EDTA). Using Dounce homogenizer, tissue was thoroughly crushed and then sonicated using a probe sonicator at 4 °C for 4 min with the following settings: 20% duty cycle, 5s pulse. The sonication cycle was then repeated for an additional 4. The lysate was transferred to a 1.5 ml Eppendorf tube and centrifuged at 4 °C for 5 min at 15,000 RPM. The supernatant collected contained active regulatory elements. The collected supernatant was treated with 2 μl RNase (50 μg/μl) and incubated at 37 °C for 10 min. Ten percent of the supernatant was transferred to fresh Eppendorf which will serve as input and the total volume was raised to 100 μl. Two microliters of proteinase K of 20 mg/ml was added to the input to remove the proteins bound with DNA and incubated at 55 °C overnight. To perform the FAIRE assay, DNA was extracted from the remaining 90% of the supernatant using the phenol-chloroform method. The DNA pellet was dissolved in 20 μl nuclease-free water and stored at −20 °C. FAIRE DNA was treated with RNase and incubated at 37 °C for 10 min. The purified DNA was extracted from both the FAIRE DNA and the input solution using phenol-chloroform and eluted to a final volume of 20 μl. DNA was quantified and 100 ng of DNA was used as a template for RT-PCR. STK38 promoter-specific primers were used for the PCR.

### Preparation of adenovirus

STK38 was cloned into pAD-CMV-DEST vector using Gateway cloning according to manufacturer protocol. Briefly, STK38 was firstly cloned into d-TOPO- Entry vector using directional cloning (Invitrogen K240020). A positive clone was selected after validating using sequencing. The positive entry clone was then cloned into p-AD-CMV- destination vector using a gateway cloning system (Invitrogen V49320). Cloned pAD-CMV-DEST-STK38 vector was then digested with PACI and transfected into 293T cells using lipofectamine 3000 (Invitrogen L3000015) for amplification. The proliferated virus was concentrated using Amicon Ultra-15 centrifugal filter (UFC901008), and approximately 10^12^ pfu/mice were administered via tail vein injection.

### Preparation of lentivirus

For STK38 knockdown, 293T cells were transfected with pLKO.1 vector containing the shRNA sequence (GCCATACCTTCGTACATGAAA) (SIGMA) for mouse STK38 along with two packaging vectors: psPAX2 and pMD2.G (Addgene). The lentiviral particles thus were generated and purified and concentrated using Amicon Ultra-15 centrifugal filter (UFC905008), and approximately 10^12^ pfu/mice were administered via tail vein injection.

### siRNA transfection

HepG2 cells were seeded on the 6-well plate and transfected with 30 nM scramble siRNA and siRNA for STK38 respectively using lipofectamine RNAiMAX (Invitrogen # 13778150) The serum-free media was replaced by complete media after 3 h of siRNA transfection. After 48 h of transfection, the cells were treated with insulin (100 nM) and incubated for 15 min. The cells were lysed and preceded for Western blot analysis.

### Western blot analysis

After treatment, cells were lysed in RIPA buffer containing 1× protease and phosphatase inhibitors and incubated on ice for 30 min with occasional vortexing. Debris was pelleted by centrifuging at 15,000 rpm for 15 min. Protein concentration was determined by the bicinchoninic acid assay reagent as described by the manufacturer’s (Thermo Fisher Scientific-23227) manual. Protein was loaded on SDS-PAGE and electroblotted onto PVDF membranes. The membrane was incubated in a 5% nonfat dry milk–blocking solution for 1 h at room temperature and probed against primary antibody (1:2000 diluted in TBST). After washing with TBST 3× for 10 min, the membrane was incubated with horseradish peroxidase–conjugated IgG secondary antibody for 2 h and visualized by chemiluminescence.

### Nuclear cytosolic fractionation

The transactivation of NF-κβ was confirmed via subcellular fractionation of the liver of RD and RD+Ad-STK38 mice following the manufacturer’s protocol. Briefly, 20 mg of liver tissue was homogenized using the NE-PER nuclear and cytoplasmic extraction kit (Thermo Fisher Scientific). The nuclear and cytoplasmic fractions were then analyzed using Western blotting.

### Coimmunoprecipitation

The immunoprecipitation assays were performed in HepG2 cells to investigate the interaction between STK38–TBK1 interactions. Firstly, TBK1 and TBK1 with STK38 were cotransfected using lipofectamine 3000 (Invitrogen). After 48 h of incubation, cells were washed two times with PBS and lysed using NP-40 lysis buffer (Hepes 30 mM, NaCl 100 mM, NP-40 0.5% (pH 7.4), Protease inhibitor) and incubated on ice for 30 min. Protein was isolated by centrifuging at 15,000 RPM for 15 min at 4 degrees. Protein was estimated using bicinchoninic acid assay kit (Thermo Fisher Scientific). Dynabeads was prewashed with PBS, and 1 mg proteins were incubated with it with an anti-HIS antibody. After incubating it overnight at 4 degrees in the spin rotor, beads were washed with washing buffer (Tris 50 mM, NaCl 100 mM, pH 7.4) three times. The proteins–antibody complex was then eluted in Laemmli buffer with 5% β-mercaptoethanol and preceded for Western blotting.

### Isolation of lipids from the liver

Snap-frozen liver samples (0.05 g) were weighed and homogenized in 10 volumes of ice-cold PBS. Forty percent of the homogenate was transferred into a fresh tube, and 1200 μl of chloroform: methanol (2:1; v/v) was added and followed by vigorous vortexing. Hundred microliters of ice-cold PBS were then added into the mixture and mixed vigorously and then centrifuged at 4200 rpm for 10 min at 4 degrees. Two hundred microliters of the organic phase were transferred into a fresh Eppendorf and evaporated for dryness in speed vac. Two hundred microliters of 1% Triton X-100 in ethanol were used to dissolve the dried lipid. TGs and cholesterol content were determined using TG (MAK266, Sigma-Aldrich) and cholesterol (AB65359, Abcam) estimation kit.

### Histopathological analysis

Mice liver tissue was fixed in 4% formaldehyde during sacrifice for cryosection and in Bouin’s solution (Sigma MFCD00146169) for Paraffin sections. Paraffin sections were prepared and stained with an H&E stain for histological analysis. To evaluate lipid accumulation, cryosection were stained with Oil Red O.

### VLDL assay

Mice fasted for 4 h after 2 h of the light cycle and 0.5 mg/g BW poloxamer was injected intraperitoneally. Blood was collected at 0, 1-, 2- ,3-, and 4-h time points, and serum TG was estimated.

### Biochemical parameters analysis

Blood was collected from the heart via cardiac puncture, and serum was isolated after centrifuging at 1000*g* for 5 min. Aspartate aminotransferase (MAK055, Sigma-Aldrich), alanine transaminase (MAK052, Sigma-Aldrich), cholesterol (AB65359, Abcam), and TGs (MAK266, Sigma-Aldrich) were estimated according to the manufacturer protocol.

### Statistical analysis

All the data presented is as mean ± SEM, assessed by Student’s *t* tests (unpaired two-tailed) or ANOVA followed by post-Bonferroni’s multiple comparisons whenever required using GraphPad Prism.

## Data availability

The datasets supporting the conclusions of this article are included within the article and its supporting information.

## Supporting information

This article contains supporting information (38, 39, 40, 41).

## Conflict of interest

No potential conflicts of interest relevant to this article were reported.
