# Supplementary material for: Diet-induced induction of hepatic serine/threonine kinase STK38 triggers proinflammation and hepatic lipid accumulation
Source: J Biol Chem. 2023 Apr 5;299(5):104678. doi: 10.1016/j.jbc.2023.104678 (PMC10193238; doi:10.1016/j.jbc.2023.104678)
Supplement: Supporting information [file mmc1.pdf]

Supporting Information

Diet-induced induction of hepatic Serine/Threonine Kinase STK38 triggers proinflammation and hepatic lipid accumulation

Priya Rawat[1], Shilpa Thakur[1], Surbhi Dogra[1], Kajal Jaswal[1], Budheswar Dehury[2], Prosenjit Mondal[1*]

[1] School of Basic Sciences, IIT Mandi, Mandi, India, [2] ICMR-Regional Medical Research Centre, Bhubaneswar, India

[*]Corresponding author

Prosenjit Mondal, PhD.,
School of Basic Sciences,
Indian Institute of Technology
Mandi, H.P -175005, India
Email: prosenjit@iitmandi.ac.in.
Phone no: (91)1950267262

Running title: hepatic STK38 triggers proinflammation and hepatic lipid accumulation.

Keywords: High fat diet; inflammation; STK38; hepatic insulin resistance; fatty liver; TBK1;

S.Fig.1.a

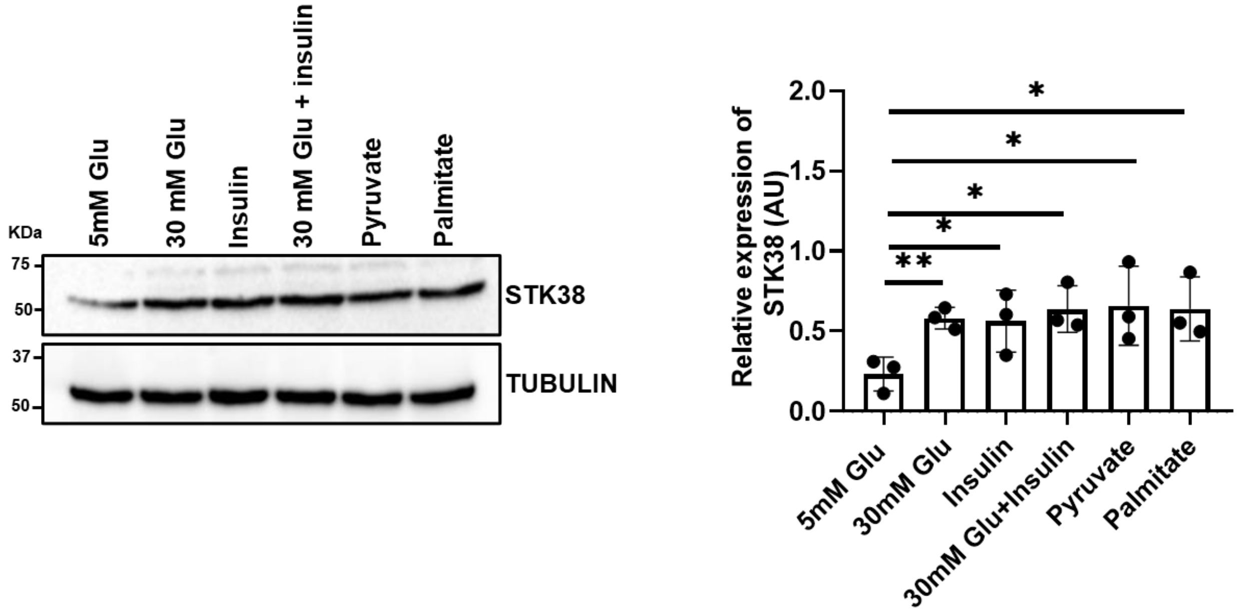

S.Fig.1. Enhanced expression of STK38 in high nutrient conditions. (a) Qualitative and quantitative expression of STK38 in different pathophysiological conditions in HepG2 cells. Mean±SEM. $*P < 0.05$, $**P < 0.01$.

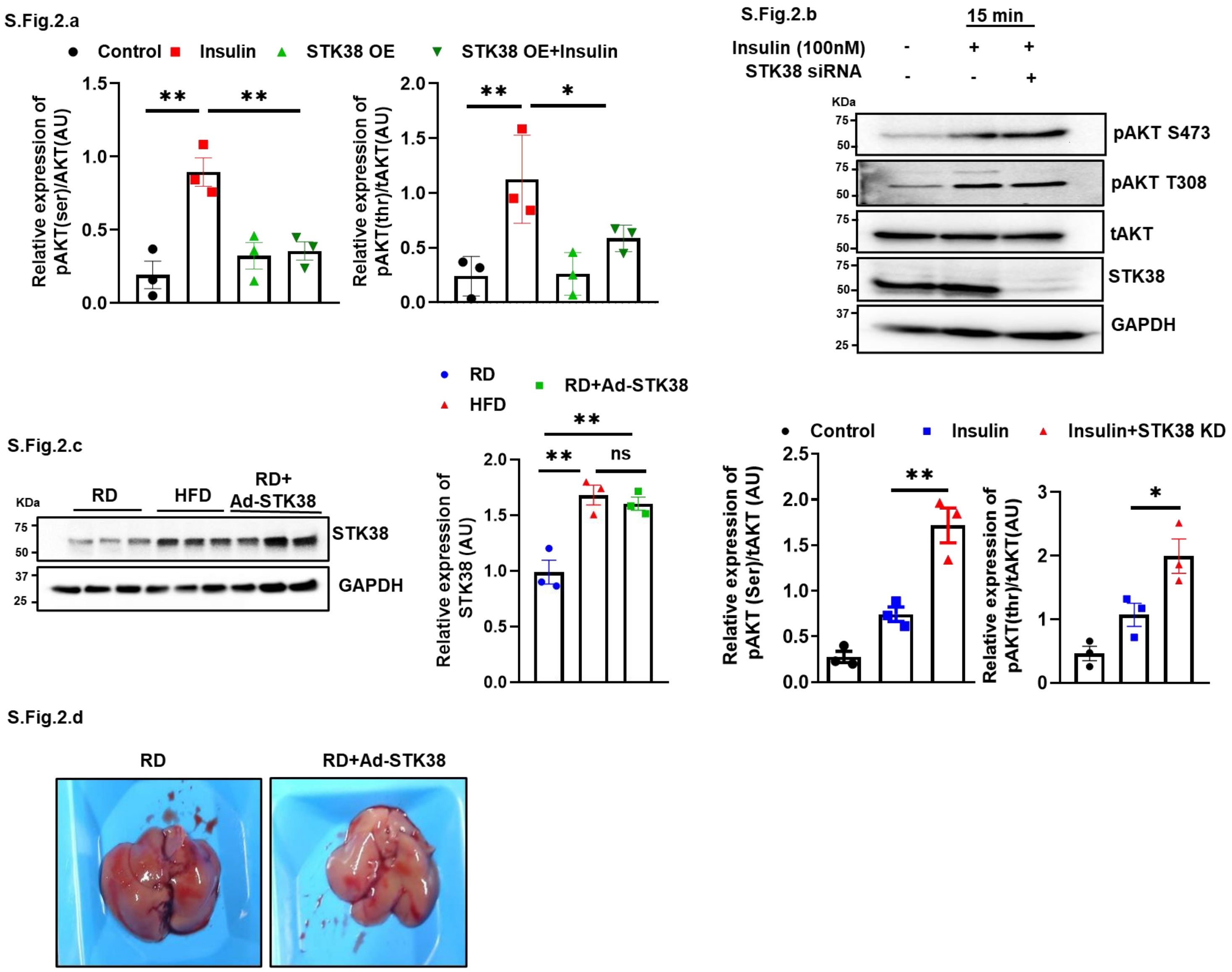

S.Fig.2. STK38 regulates hepatic insulin signaling. **(a)** Quantitative expression of phospho-AKT at ser473 and thr308 after STK38 overexpression. **(b)** Qualitative and quantitative expression of phospho-AKT at ser473 and thr308 after STK38 knockdown. **(c)** Qualitative and quantitative expression of STK38 in RD, HFD, RD+Ad-STK38 mice. **(d)** Liver of RD and RD+Ad-STK38. Mean±SEM. *$P < 0.05$, **$P < 0.01$.

Supporting Information

S.Fig.3.a

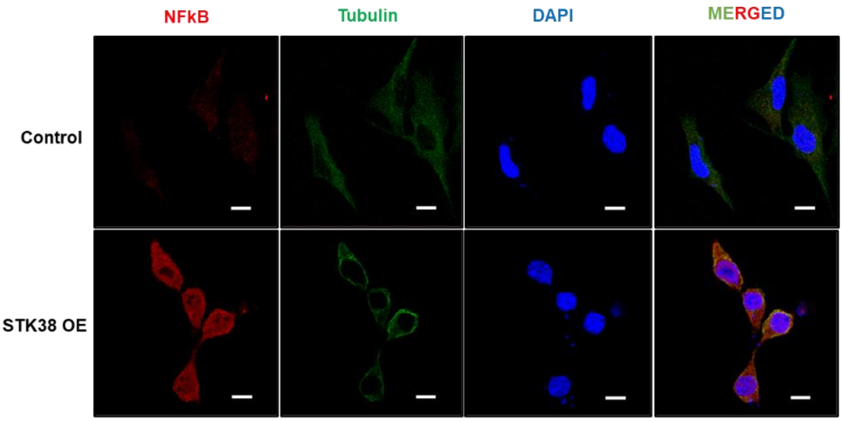

S.Fig.3.b

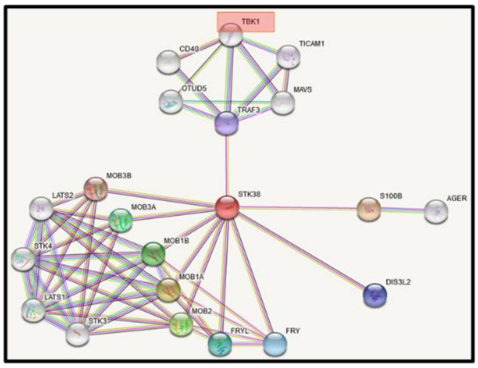

S.Fig.3.C

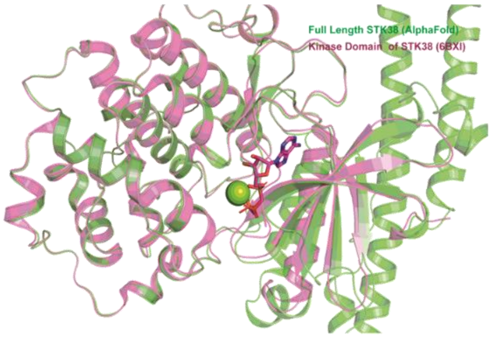

S.Fig.3.d

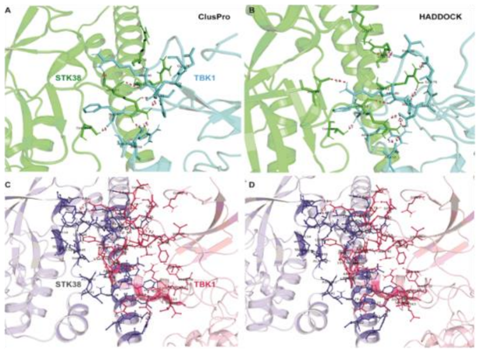

S.Fig.3.e

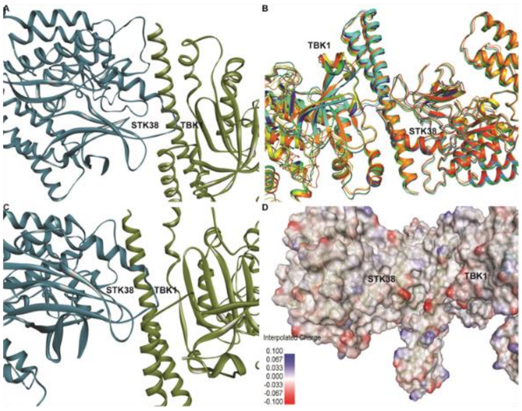

S.Fig.3.f

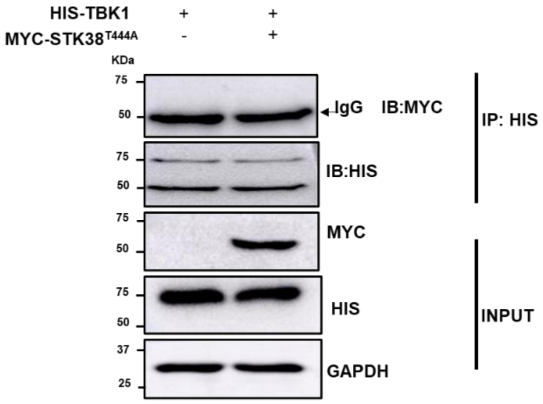

S.Fig.3.g

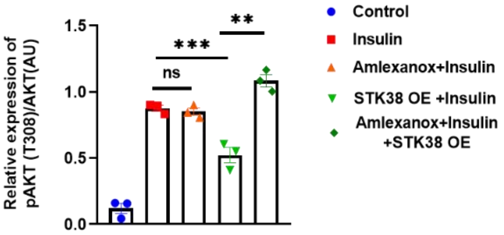

S.Fig. 3. STK38 potentiates NFKB transactivation via TBK1. (a) Nuclear translocation of NF-Kβ after 48hrs of STK38 overexpression in HepG2 was observed after ICC. Scale 20µm. **(b)** String.org map of STK38 showing TBK1 as a predicted interacting partner. **(c)** Structural superimposed view of the x-ray crystal structure of NDR1 kinase domain (PDB ID: 6BXI) of STK38 (magenta) the ligand ANP and Mg^{+2} and full-length model (green) derived from Alpha-Fold derived using PyMOL. **(d)** Intermolecular contact analysis displaying important non-bonded contacts formed between STK38 and TBK1 predicted by ClusPro, HADDOCK, and PRODIGY**. (d.A)** Top-ranked pose of the protein-protein complexes of STK38 and TBK1 obtained from ClusPro. **(d.B)** Top-ranked pose of the protein-protein complexes of STK38 and TBK1 obtained from HADDOCK. **(d.C)** PRODIGY analysis displaying the interfacial contacts formed between wild-type STK38 and TBK1. **(d.D)** PRODIGY analysis displaying the interfacial contacts formed between mutant type STK38 (T444A) and TBK1. **(e)** Top scored conformational states of the STK38 with TBK1 from ClusPro and HADDOCK. **(e.A)** Zoomed-in-view the top-scored pose of STK38 with TBK1 predicted by ClusPro. **(e.B)** Superimposed zoomed-in-view all the top clusters of STK38 with TBK1 predicted by HADDOCK. **(e.C)** Zoomed-in-view the top-scored pose of STK38 with TBK1 predicted by HADDOCK. **(e.D)** Interpolated charges patches were assessed using the top-scored pose of STK38 with TBK1 predicted by HADDOCK. **(f)** MYC-$STK38^{T444A}$ mutant and HIS-TBK1 mutant were overexpressed in HepG2 cells and CO-IP was performed using Anti-HIS-Ab and immunoblotted with MYC and HIS. **(g)** quantitative expression of phospho-AKT thr308 after pharmacological inhibition of TBK1 in HepG2 cells. Mean±Sem. $^{**}P < 0.01$, $^{***}P < 0.001$

S.Fig.4.a

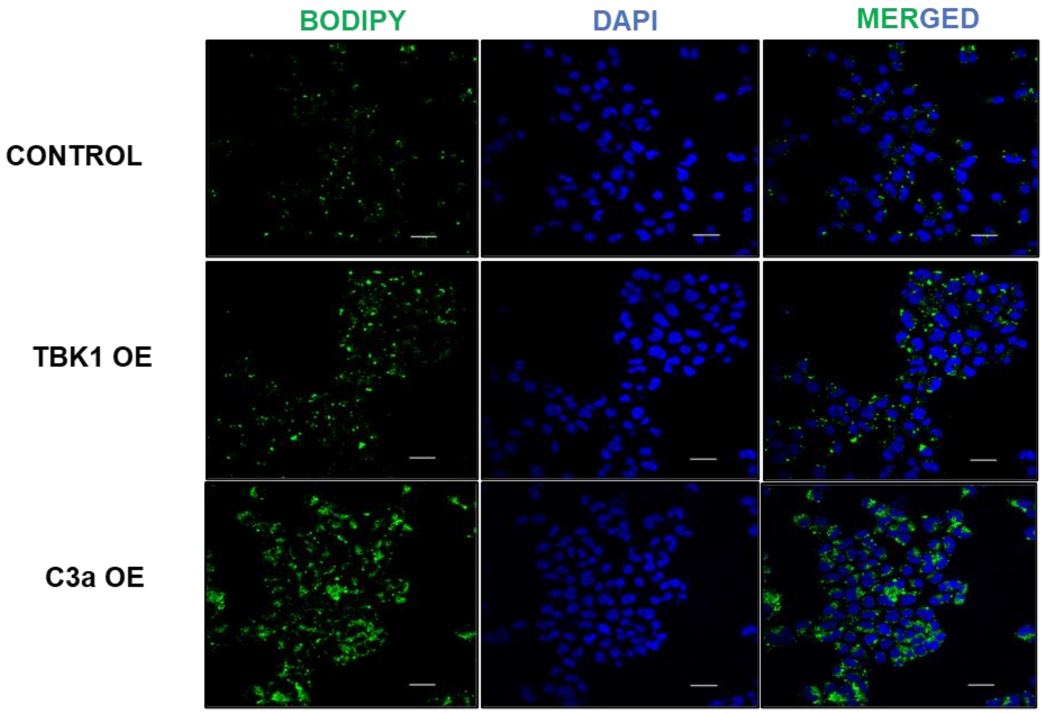

S.Fig.4. STK38 exacerbates hepatic lipid accumulation via TBK1 and C3a activation. BODIPY staining in HepG2 cells after TBK1 and C3a overexpression. Scale 20µm.

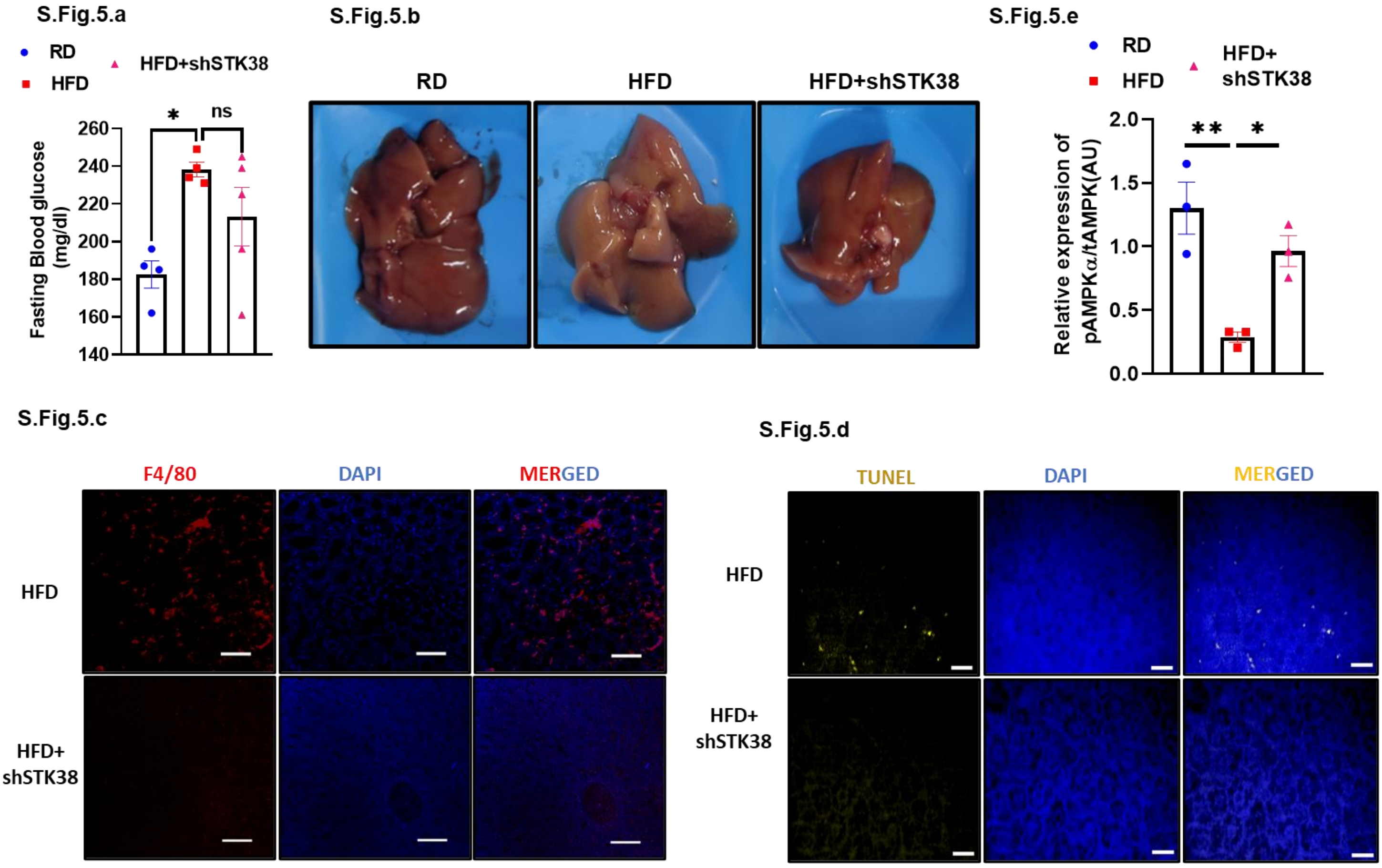

S.Fig.5. STK38 knockdown improves high-fat diet-mediated hepatic inflammation and lipid accumulation (a) Fasting blood glucose in RD, HFD, and HFD+shSTK38 mice after 6 hrs of fasting. **(b)** liver of RD, HFD, HFD+shSTK38 **(c)** F4/80 staining in liver of HFD and HFD+shSTK38. Red fluorescence F4/80 and blue fluorescence DAPI. Scale 20µm. **(d)** Tunel staining in liver tissue. Yellow fluorescence tunel stain and blue fluorescence DAPI. Scale 20µm. **(e)** Quantitative representation of AMPK in the liver of RD, HFD, and HFD+shSTK38. Mean±Sem. *$P < 0.05$, **$P < 0.01$.

Supporting Information

S.no.	Accession	Description
1	P15864	Histone H1.2 OS=Mus musculus OX=10090 GN=H1-2 PE=1 SV=2
2	P43274	Histone H1.4 OS=Mus musculus OX=10090 GN=H1-4 PE=1 SV=2
3	P43277	Histone H1.3 OS=Mus musculus OX=10090 GN=H1-3 PE=1 SV=2
4	P43276	Histone H1.5 OS=Mus musculus OX=10090 GN=H1-5 PE=1 SV=2
5	Q8VDD5	Myosin-9 OS=Mus musculus OX=10090 GN=Myh9 PE=1 SV=4
6	P08730	Keratin, type I cytoskeletal 13 OS=Mus musculus OX=10090 GN=Krt13 PE=1 SV=2
7	Q60605	Myosin light polypeptide 6 OS=Mus musculus OX=10090 GN=Myl6 PE=1 SV=3
8	Q05920	Pyruvate carboxylase, mitochondrial OS=Mus musculus OX=10090 GN=Pc PE=1 SV=1
9	Q64523	Histone H2A type 2-C OS=Mus musculus OX=10090 GN=H2ac20 PE=1 SV=3
10	P05784	Keratin, type I cytoskeletal 18 OS=Mus musculus OX=10090 GN=Krt18 PE=1 SV=5
11	Q9CXS4	Centromere protein V OS=Mus musculus OX=10090 GN=Cenpv PE=1 SV=2
12	Q5U405	Transmembrane protease serine 13 OS=Mus musculus OX=10090 GN=Tmprss13 PE=2 SV=2
13	Q9WV98	Mitochondrial import inner membrane translocase subunit Tim9 OS=Mus musculus OX=10090 GN=Timm9 PE=1 SV=1
14	Q9R1K9	Centrin-2 OS=Mus musculus OX=10090 GN=Cetn2 PE=1 SV=1
15	P01027	Complement C3 OS=Mus musculus OX=10090 GN=C3 PE=1 SV=3
16	P10922	Histone H1.0 OS=Mus musculus OX=10090 GN=H1-0 PE=2 SV=4
17	Q8BFU3	RING finger protein 214 OS=Mus musculus OX=10090 GN=Rnf214 PE=1 SV=1
18	P14148	60S ribosomal protein L7 OS=Mus musculus OX=10090 GN=Rpl7 PE=1 SV=2
19	O55142	60S ribosomal protein L35a OS=Mus musculus OX=10090 GN=Rpl35a PE=1 SV=2
20	P62908	40S ribosomal protein S3 OS=Mus musculus OX=10090 GN=Rps3 PE=1 SV=1

Supporting Table 1. List of proteins found in LC-MS after performing DNA pull-down assay. Potentially interacting protein binding to the promoter of STK38.

Pairs	Distance	Type	Category
**A:ARG83:HH21 - B:ASP50:OD2**	1.90	Hydrogen Bond;Electrostatic	Salt Bridge
**A:ARG83:HH22 - B:GLU75:OE2**	1.83	Hydrogen Bond;Electrostatic	Salt Bridge
**B:ARG80:HH11 - A:GLU87:OE1**	1.76	Hydrogen Bond;Electrostatic	Salt Bridge
**B:ARG80:HH21 - A:ASP88:OD2**	2.03	Hydrogen Bond;Electrostatic	Salt Bridge
**A:ARG19:HE - B:GLU55:OE1**	1.98	Hydrogen Bond	Conventional Hydrogen Bond
**A:ARG19:HH21 - B:ARG54:O**	2.86	Hydrogen Bond	Conventional Hydrogen Bond
**A:TYR31:HH - B:ARG162:O**	1.84	Hydrogen Bond	Conventional Hydrogen Bond
**A:ASN33:HD21 - B:LEU46:O**	1.91	Hydrogen Bond	Conventional Hydrogen Bond
**A:ARG78:HE - B:THR78:O**	2.04	Hydrogen Bond	Conventional Hydrogen Bond
**A:ARG78:HH22 - B:THR78:O**	1.81	Hydrogen Bond	Conventional Hydrogen Bond
**A:ARG81:HH11 - B:THR79:OG1**	1.81	Hydrogen Bond	Conventional Hydrogen Bond
**A:ARG83:H - B:ARG80:O**	2.75	Hydrogen Bond	Conventional Hydrogen Bond
**A:THR444:HG1 - B:PHE45:O**	1.81	Hydrogen Bond	Conventional Hydrogen Bond
**B:SER44:H - A:LYS80:O**	2.75	Hydrogen Bond	Conventional Hydrogen Bond
**B:SER44:HG - A:ARG81:O**	1.87	Hydrogen Bond	Conventional Hydrogen Bond
**B:PHE45:H - A:LYS80:O**	2.12	Hydrogen Bond	Conventional Hydrogen Bond
**B:ARG54:HH21 - A:MET22:SD**	2.48	Hydrogen Bond	Conventional Hydrogen Bond
**B:ARG80:HH12 - A:ARG83:O**	2.43	Hydrogen Bond	Conventional Hydrogen Bond
**A:LEU84:H - B:PHE45**	3.14	Hydrogen Bond	Pi-Donor Hydrogen Bond

Supporting Table 2. Analysis of the top-ranked cluster of protein-protein docked complex obtained from ClusPro displaying various non-bonded intermolecular contacts formed between STK38 (chain A) and TBK1 (chain B). key interacting residues are highlighted

Supporting Information

Interacting Pairs	Distance	Type	Category
**A:ARG19:HH22 - B:GLU55:OE1**	1.55	Hydrogen Bond;Electrostatic	Salt Bridge;Attractive Charge
**A:ARG81:HH22 - B:GLU76:OE1**	1.92	Hydrogen Bond;Electrostatic	Salt Bridge;Attractive Charge
**A:ARG83:HH11 - B:ASP50:OD2**	1.79	Hydrogen Bond;Electrostatic	Salt Bridge;Attractive Charge
**A:ARG83:HH21 - B:GLU75:OE1**	1.77	Hydrogen Bond;Electrostatic	Salt Bridge;Attractive Charge
**A:ARG83:HH22 - B:ASP50:OD2**	1.87	Hydrogen Bond;Electrostatic	Salt Bridge;Attractive Charge
**B:ARG80:HH12 - A:GLU87:OE1**	1.56	Hydrogen Bond;Electrostatic	Salt Bridge;Attractive Charge
**B:ARG80:HH21 - A:ASP88:OD2**	2.58	Hydrogen Bond;Electrostatic	Salt Bridge;Attractive Charge
**B:ARG80:HH22 - A:GLU87:OE1**	2.72	Hydrogen Bond;Electrostatic	Salt Bridge;Attractive Charge
**B:ARG80:HH22 - A:ASP88:OD2**	2.59	Hydrogen Bond;Electrostatic	Salt Bridge;Attractive Charge
**A:ARG19:NH1 - B:GLU55:OE2**	5.21	Electrostatic	Attractive Charge
**A:ARG83:NH1 - B:GLU75:OE2**	4.91	Electrostatic	Attractive Charge
**A:ARG83:NH2 - B:ASP50:OD1**	3.64	Electrostatic	Attractive Charge
**A:ARG19:HE - B:GLU55:OE1**	2.76	Hydrogen Bond	Conventional Hydrogen Bond
**A:TYR31:HH - B:ARG162:O**	2.15	Hydrogen Bond	Conventional Hydrogen Bond
**A:ARG78:HE - B:THR78:O**	1.75	Hydrogen Bond	Conventional Hydrogen Bond
**A:ARG78:HH22 - B:THR78:O**	2.59	Hydrogen Bond	Conventional Hydrogen Bond
**A:ARG81:HH12 - B:PHE40:O**	2.71	Hydrogen Bond	Conventional Hydrogen Bond
**A:ARG81:HH12 - B:HIS81:ND1**	2.29	Hydrogen Bond	Conventional Hydrogen Bond
**A:ARG81:HH22 - B:GLN2:OE1**	3.06	Hydrogen Bond	Conventional Hydrogen Bond
**A:ARG83:HN - B:ARG80:O**	2.31	Hydrogen Bond	Conventional Hydrogen Bond
**A:ARG83:HE - B:GLU75:OE2**	1.98	Hydrogen Bond	Conventional Hydrogen Bond
**A:THR444:HG1 - B:PHE45:O**	1.73	Hydrogen Bond	Conventional Hydrogen Bond
**A:THR444:HG1 - B:ARG47:O**	3.02	Hydrogen Bond	Conventional Hydrogen Bond
**B:SER44:HG - A:ARG81:O**	2.03	Hydrogen Bond	Conventional Hydrogen Bond
**B:PHE45:HN - A:LYS80:O**	2.22	Hydrogen Bond	Conventional Hydrogen Bond
**B:ARG54:HH11 - A:MET22:SD**	2.12	Hydrogen Bond	Conventional Hydrogen Bond
**B:ARG54:HH21 - A:MET22:SD**	2.86	Hydrogen Bond	Conventional Hydrogen Bond
**B:LYS197:HZ1 - A:SER13:OG**	2.00	Hydrogen Bond	Conventional Hydrogen Bond
**A:LYS446:NZ - B:PHE45**	4.52	Electrostatic	Pi-Cation
**B:ARG47:NH1 - A:PHE30**	4.97	Electrostatic	Pi-Cation
**B:ARG162:NH1 - A:PHE30**	3.74	Electrostatic	Pi-Cation
**B:ARG162:NH1 - A:TYR31**	3.64	Electrostatic	Pi-Cation
**A:THR444:CG2 - B:PHE45**	3.99	Hydrophobic	Pi-Sigma
**B:LEU164:CD1 - A:TYR31**	3.73	Hydrophobic	Pi-Sigma
**A:LYS80 - B:ILE43**	3.91	Hydrophobic	Alkyl
**A:ARG81 - B:ILE43**	5.46	Hydrophobic	Alkyl
**A:ARG83 - B:ARG80**	4.30	Hydrophobic	Alkyl
**B:PRO48 - A:ILE441**	4.31	Hydrophobic	Alkyl
**A:PHE30 - B:ARG47**	3.99	Hydrophobic	Pi-Alkyl
**A:TYR31 - B:ARG162**	4.69	Hydrophobic	Pi-Alkyl
**B:PHE45 - A:LYS446**	4.42	Hydrophobic	Pi-Alkyl
**B:HIS81 - A:ARG81**	4.66	Hydrophobic	Pi-Alkyl

Supporting Table 3. Analysis of the top-ranked cluster of protein-protein docked complex obtained from HADDOCK displaying various non-bonded contacts formed between STK38 (chain A) and TBK1 (chain B). key interacting residues are highlighted.

Protein-protein complex	ΔG (kcal mol^{-1})	K_d (M) at 25.0 ℃	Number of Interfacial Contacts (ICs)
**WT (STK38-TBK1)**	-10.9	1.1E-08	ICs charged-charged: 13<br>ICs charged-polar: 19<br>ICs charged-apolar: 24<br>ICs polar-polar: 5<br>ICs polar-apolar: 15<br>ICs apolar-apolar: 18
**MT (T444A STK38-TBK1)**	-10.2	3.2E-08	ICs charged-charged: 13<br>ICs charged-polar: 18<br>ICs charged-apolar: 25<br>ICs polar-polar: 4<br>ICs polar-apolar: 11<br>ICs apolar-apolar: 21

Supporting Table 4. Analysis of Binding affinity of STK38 (WT and Mutant: T444A) and TBK1 using PRODIGY. The top-ranked cluster obtained from HADDOCK was used for binding affinity prediction.

Material and methods

DNA pull-down assay

DNA pull-down assay was performed as described by Wang et al.[1] In brief, Biotinylated primers for STK38 promoter were designed from +29- -600. The promotor fragment was amplified using PCR and the product was immobilized on neutravidin beads with DNA binding buffer (TRIS (pH 7.5)-5mM/L, EDTA -O.5mM/L, NaCl- 1 mM/L). Washing was performed with BC-150 buffer (TRIS (pH 7.3)-20mM/L, EDTA -O.2mM/L, NaCl- 150 mM/L, Glycerol-20%). Nuclear proteins were extracted using NE-PER Nuclear and Cytoplasmic Extraction Reagents (Thermo) containing Halt protease inhibitor cocktail. Before adding STK38 promotor- neutravidin complex, the nuclear extract was adjusted to 200–250 mmol/L total salt with BC-0(TRIS (pH 7.3)-20mM/L, EDTA -O.2mM/L, Glycerol-20%, EGTA/EDTA-1 mmol/L). STK38 promoter- neutravidin was incubated with 250ug nuclear extract at 4°C overnight. The supernatant was discarded and the beads were washed twice with NETN buffer (NaCl -50 mmol/L, EDTA-1 mmol/L, Tris [pH 8.0]- 20 mmol/L, NP-40- 0.5%) & Three times with PBS. Beads were resuspended in 50µL sample buffer, boiled for 5 min, and LC-MS was performed.

SDM

To develop STK38 T444A mutant, a gene-specific primer with a mutated site was designed using NEB base changer, and mutagenesis was done using Q5 site-directed mutagenesis kit from NEB (Cat E0554) as per the manufacturer's guideline.

Co-Immunoprecipitation

The immunoprecipitation assays were performed in HepG2 cells to investigate the interaction between STK38 T444A-TBK1 interaction. TBK1 and TBK1 with STK38 T444A were co-transfected using lipofectamine 3000 (Invitrogen). After 48 hours of incubation cells were washed two times with PBS and lysed using NP-40 lysis buffer (HEPES 30mM, NaCl 100mM, NP-40 0.5% (pH 7.4), Protease inhibitor) and incubated on ice for 30 mins. Protein was isolated by centrifuging at 15000 RPM for 15mins at 4 degrees. Protein was estimated using BCA assay kit (Thermo Scientific). Dynabeads^{TM} was pre-washed with PBS and 1mg proteins were incubated with it with anti-HIS antibody. After incubating it overnight at 4 degrees in the spin rotor, beads were washed with washing buffer (TRIS 50mM, NaCl 100mM, pH 7.4) 3 times. The proteins-antibody complex was then eluted in Laemilli buffer with 5% BME and proceeded for western blotting.

Protein-protein docking of STK38 with TBK1

Herein, to understand the plausible mode of protein-protein interaction mediated by STK38 and TBK1, we used the full-length structure of STK38 from the Alpha Fold database (which perfectly superpose with the experimental kinase domain (PDB ID: 6BXI) for docking with experimentally solved TBK2 (PDB ID:4IM0). Both the proteins were prepared by optimization of hydrogen bonds and the addition of missing hydrogen atoms with PROPKA3.0 at pH 7.0 using the Protein preparation wizard of Maestro12.8 (Schrödinger, LLC, New York, 2021-2). The optimized proteins were submitted to the state-of-the-art tool ClusPro for protein-protein docking with default parameters.[2] The top-ranked representative cluster (having a higher number of structures with a higher weighted score) obtained from ClusPro was subjected to refinement using High Ambiguity Driven protein-protein Docking (HADDOCK), where, interacting residues obtained were provided as active site residues for flexible docking of biomolecular complexes.[3] The top cluster with a higher HADDOCK Z-score (negative) was retained further for non-bonded contact analysis.

Binding Affinity Assessment

To assess the importance of T444 of STK38 in molecular recognition of TBK1, we computationally designed an alanine mutant T444A using the mutagenesis tool employed in PyMOL and computed the binding affinity (ΔG) of wild type and mutant type of STK38 with TBK1. We have employed PRODIGY, a tool that uses contact-based prediction of binding affinity in protein-protein complexes.[4]

Immunocytochemistry

HepG2 Cells were seeded on coverslips precoated with poly-L-lysine. After 48 hrs of transfection, cells were washed with PBS and fixed with 4% formaldehyde at RT. Further, cells were washed with PBST (0.05% Tween 20 in PBS). Cells were blocked using a 2% FBS blocking buffer prepared in PBST and allowed to shake moderately for 1 h at RT. Cells were incubated with primary antibody (1:300 dilution) overnight at 4°C. After washing with PBST (0.05% Tween-20), cells were incubated with Alexa-Fluor Secondary antibodies at 1:1000 dilution for 2 hrs. Cells were washed 3 times with PBST and mounted with DAPI mounting media (company). The images were recorded by using confocal (Nikon) microscopy.

Immunohistochemistry

Bouin's fixed liver tissue was embedded in paraffin and 5µm sections were made, deparaffinized, rehydrated, and washed with 1x PBS. Antigen retrieval was performed by microwaving the slide for 15 min with sodium citrate buffer (ph 6). Slides were washed and permeabilized with 0.1 percent triton-X in PBS. Tissue was blocked with 2% Neonatal horse serum for 1 hour and incubated with 1:300 dilution F4/80 (CST) overnight at 4 degrees. The next day, the Slides were washed with PBST and incubated with 1:1000 dilution secondary antibody (Alexa-647) for 2 hr and mounted with DAPI mounting media. Imaging was done using a confocal microscope (Nikon).

Bodipy staining

Cells were plated on coverslips and after 48hrs of transfection, cells were fixed with 4 % formaldehyde and washed 3 times with PBS, and stained with 2µM BODIPY493/503 (Invitrogen) and incubated at 37 degrees Celsius for 30 mins. Cells were washed 3x with PBS and mounted with DAPI mounting media. lipid droplets were analyzed using confocal microscopy (Nikon).

Dead-end Tunel assay

Tunel staining was performed as per the manufacturer's protocol. In brief, bouin's fixed liver tissue was embedded in paraffin and 5µm sections were made, deparaffinized, rehydrated, and washed with 1x PBS. Antigen retrieval was performed by using proteinase K (20µg/ml) and proceeded for TdT labeling. Slides were mounted with DAPI mounting media and analyzed using a confocal microscope (Nikon).

Reference

1. Wang, Liheng, Junjie Yu, Qiuzhong Zhou, Xiaobo Wang, Maria Mukhanova, Wen Du, Lei Sun, Utpal B. Pajvani, and Domenico Accili. "TOX4, an Insulin Receptor-Independent Regulator of Hepatic Glucose Production, Is Activated in Diabetic Liver." *Cell Metabolism* 34, no. 1 (January 4, 2022): 158-170.e5.

2. Kozakov, Dima, David R. Hall, Bing Xia, Kathryn A. Porter, Dzmitry Padhorny, Christine Yueh, Dmitri Beglov, and Sandor Vajda. "The ClusPro Web Server for Protein-Protein Docking." *Nature Protocols* 12, no. 2 (February 2017): 255–78.

3. Zundert, G. C. P. van, J. P. G. L. M. Rodrigues, M. Trellet, C. Schmitz, P. L. Kastritis, E. Karaca, A. S. J. Melquiond, M. van Dijk, S. J. de Vries, and A. M. J. J. Bonvin. "The HADDOCK2.2 Web Server: User-Friendly Integrative Modeling of Biomolecular Complexes." *Journal of Molecular Biology* 428, no. 4 (February 22, 2016): 720–25.

4. "PRODIGY: A Web Server for Predicting the Binding Affinity of Protein–Protein Complexes | Bioinformatics | Oxford Academic." Accessed January 2, 2023.

Chemicals:

S.No.	Chemical	Company	Cat. No.
1.	Amlexanox	Abcam	ab142825
2.	iScript cDNA synthesis kit	Biorad	170881
3.	iTaq Universal SYBR Green Supermix	Biorad	1725121
4.	Insulin	Biorad	10516
6.	gateway cloning system	Invitrogen	V49320
7.	lipofectamine 3000	Invitrogen	L3000015
8.	lipofectamine RNAiMAX	Invitrogen	13778150
9.	BCA assay	Thermo Scientific	23227
10.	Nuclear and cytoplasmic extraction kit	Thermo Scientific	78833
11	ORO	Sigma	O0625-25G
12	Bouin's solution	Sigma	MFCD00146169
13	Q5® Site-Directed Mutagenesis Kit	NEB	E0554S
14	Bodipy 493/503	Thermo Scientific	D3922
15	DeadEnd™ Fluorometric TUNEL System	Promega	G3250
16	Fluoroshield™ with DAPI	Sigma	F6057-20ML
17	Amicon Ultra-15 centrifugal filter	Merck	UFC901008
18	Amicon Ultra-15 centrifugal filter	Merck	UFC905008

Antibodies:

S.No.	Antibody	Company	Cat. No.
1.	pAKT S473	CST	4058S
2	pAKT T308	CST	4056
3.	tAKT	CST	9272s
4.	pGSK3β	CST	9323s
5	tGSK3β	CST	9315
6.	STK38	ABNOVA	H00011329M02
7.	pTBK1	CST	5483
8.	tTBK1	CST	3013
9.	tNF-κβ	CST	6956
10.	pAMPKα	CST	2535s
11.	tAMPKα	CST	2535
12.	pACC	CST	3661
13.	tACC	CST	3676
14.	GAPDH	CST	d16h11(5174)
15.	α −Tubulin	CST	2144
16.	Myc-tag	CST	2278
17.	F4/80	CST	70076
18.	Alexa-647-anti-mouse IgG	Jackson ImmunoResearch	115-605-006

Supporting Information

Primers:

**Gene**	**Species**	**Sequence**
TOPO-STK38 FP	Human	CACCATGGCAATGACAGGCTCAAC
TOPO-STK38 RP	Human	CTATTTTGCTGCTTTCATGTAGGAAG
QPCR-C3a FP	Mouse	GGAAGTGTTGTGAGGATGGTAT
QPCR-C3a RP	Mouse	GTGGTTGCAGCAGTCTATGA
5'biosig STK38 Promotor FP	Mouse	AAATCAATGAGAGGCTTTAAGTTG
5'biosig STK38 Promotor RP	Mouse	ACCCCCTCAGACTGTGGA
C3A AAV-MCS ECOI FP	Mouse	CGGAATTCATGTCAGTACAGTTGATGGAAAGAA
C3A AAV-MCS HindII RP	Mouse	CCCAAGCTTCTACCTGGCCAGGCCCAGCAC
STK38 T444A SDM FP	Human	AAGACCCAGTCTTTGTTCTTG
STK38 T444A SDM RP	Human	AAATCAATGAGAGGCTTTAAGTTG
STK38 promotor -300-1 FP	Mouse	GTAAGACCTTGAGGGAAGCTG
STK38 promotor -300-1 RP	Mouse	GCTGGCCTTTGTTATGTTACTTT

S.No.	Plasmid	Cat.no,
1.	STK38-myc	HG12319-NM
2.	TBK1-his	HG11023-CH
3	pLKO.1-STK38	TRCN0000022864
4.	Empty Vector pLKO.1	SHC001
